# Evaluating a Multimedia Messaging Intervention to Increase Adolescent HIV Testing: Protocol for a Hybrid Type 1 Effectiveness Implementation Study

**DOI:** 10.2196/88465

**Published:** 2026-05-18

**Authors:** Kathryn Macapagal, Andrés Alvarado Avila, Zach M Buehler, Jacob Gordon, Ashley Knapp, Manuel Hurtado Jr, Julianna Lorenzo, James Foran, Michael E Newcomb, Brian Mustanski, Dennis H Li, Gregory Swann, Mariajosé J Paton, Rana Saber, Elizabeth Casline

**Affiliations:** 1 Department of Medical Social Sciences, Feinberg School of Medicine, Northwestern University, 625 N Michigan Ave, Suite 1400, Chicago, IL, 60611, United States, 1 312-503-3605; 2 Department of Psychiatry and Behavioral Sciences, Feinberg School of Medicine, Northwestern University, Chicago, IL, United States; 3 Impact Institute, Northwestern University, Chicago, IL, United States; 4 College of Nursing, University of Cincinnati, Cincinnati, OH, United States

**Keywords:** mHealth, digital health, multimedia messaging, adolescent, HIV testing, sexual and gender minority, implementation

## Abstract

**Background:**

Sexual and gender minority (SGM) adolescents who can benefit from HIV testing due to their sexual behavior (eg, condomless anal or vaginal sex) report suboptimal HIV testing rates. However, interventions designed to increase HIV testing rates among SGM adolescents are lacking, and even when effective interventions exist, reaching SGM adolescents outside research settings can be a challenge. This hybrid type 1 study aims to evaluate the effectiveness of Sharing Health Education Resources (SHER), a multimedia messaging service (MMS) intervention in increasing adolescent HIV testing rates and to address implementation challenges of reaching and engaging SGM adolescents in digital interventions in real-world settings.

**Objective:**

This study first aims to adapt a digital sexual health intervention previously designed for adolescent sexual minority males to be inclusive of all SGM adolescents and reflect new advancements in HIV prevention science. The second aim is to test the adapted intervention through a hybrid type-1 effectiveness-implementation randomized controlled trial (RCT) with the primary outcome of HIV testing. The third aim is to gather expert recommendations on how to best reach and engage SGM adolescents outside of research settings.

**Methods:**

SHER is guided by the information-motivation-behavioral skills theory and expands upon a previous intervention tested in a 2014 pilot trial. The previous version of the intervention significantly increased self-reported HIV testing among adolescent sexual minority males compared to an information-only control. Updates to this intervention in this study were driven by stakeholder and adolescent feedback and included modernizing technology, content, and gender inclusivity. An RCT will compare the impact of SHER versus an information-only control condition on HIV testing rates among 360 US SGM adolescents aged 13‐19 years. The implementation aim is focused on understanding expert and adolescent perspectives on best practices for reaching and engaging SGM adolescents outside of research settings.

**Results:**

Data collection regarding intervention updates was concluded in January 2025 with publication of results planned in fall 2026. As of April 2026, 295 participants have been enrolled into the RCT, with the conclusion of data collection, analysis, and publication of results planned for Winter 2027. All data collection regarding best practices for reaching and engaging SGM adolescents outside of research settings was completed in September 2025, with publication of results planned for Summer 2026.

**Conclusions:**

This study will evaluate whether an MMS messaging intervention based on health behavior change theory will increase SGM adolescent HIV testing relative to an information-only version of this intervention. This study will also produce expert- and adolescent-informed best practices for reaching SGM adolescents for sexual health interventions in real-world settings. Overall, this study aims to contribute to increased engagement in sexual health care and HIV testing in SGM adolescents engaging in HIV transmission risk behaviors.

## Introduction

In the United States, an estimated 44% of youth aged 13‐24 years who are HIV-positive are unaware of their status, making this the group with the highest proportion of undetected HIV infections across all age brackets [1-3]. Even though clinical and public health recommendations suggest screening for HIV at least once between the ages of 15 years and 21 years [4], yearly for those engaging in HIV transmission risk behavior (eg, condomless penetrative sex without HIV pre-exposure prophylaxis [PrEP]) [5], and every 3-6 months for sexually active men who have sex with men [5], testing rates do not reflect these guidelines [6]. This issue is particularly pronounced among sexual and gender minority (SGM) adolescents, with only 1 in 4 gay and bisexual teenage boys [7], approximately 1 in 7 transgender adolescents [8], and variable rates among sexual minority girls, ranging from just 1 in 40 girls who have sex with girls only to 1 in 4 girls who have sex with both boys and girls [9] reporting HIV testing. However, programs aimed at increasing HIV testing predominantly focus on adults and heterosexual, cisgender youth [10-13], which may not address the unique barriers faced by SGM adolescents, such as fear of being outed, anticipated stigma and discrimination in health care settings, and developmental concerns about navigating sexual identity while accessing sexual health services.

Digital interventions delivered via smartphones offer a promising strategy for reaching SGM adolescents, as 95% of adolescents report using smartphones and rely on texting to communicate [14]. Compared to app-based interventions, those delivered via multimedia messaging service (MMS) are more accessible, scalable, and easy to use [15-19]. Further, they can be more cost-efficient, require minimal data, and perform reliably across operating systems. These features make MMS-based interventions well-suited to addressing structural barriers, including transportation limitations, restrictive clinic hours, geographic isolation, financial constraints, or confidentiality concerns, that often limit access to in-person and web-based services, which disproportionately affect Black and Latino youth and those in lower-resourced communities [20-22].

Despite these advantages, few studies have explored considerations for implementing MMS-based HIV prevention programs in real-world settings [23,24]. In an ever-changing sociotechnical environment, digital interventions designed to be tested in research studies (and not necessarily with real-world implementation in mind) may change little over the course of several years. These interventions often fail to keep up with real-world user expectations of technology and design and thus seem quickly dated. In addition, in fields like sexual health and HIV, new innovations occur often and necessitate changes to intervention content to remain medically accurate. Moreover, it is not enough to build an effective digital intervention and leave it on the shelf. Implementers must be able to successfully reach and engage desired users once the intervention is deployed in the real world. For digital interventions on sensitive topics like adolescent sexual health to succeed, it is critical to reach and build trust with youth where they already are (ie, online). However, this task can be daunting for organizations who seek to engage SGM adolescents, as the online spaces where SGM adolescents spend their time change quickly, and advertising to or reaching minors can be expensive, and platforms’ policies regarding advertising can be opaque or change often, which can constrain organizations’ ability to reach this group.

Studies that examine both how well digital HIV prevention and testing interventions for adolescent populations work and how to overcome real-world implementation challenges like reaching SGM adolescents are needed to address the domestic HIV epidemic. In response, this paper describes the protocol for a type 1 hybrid study to evaluate the effectiveness and implementation of an adapted version of Guy2Guy (G2G), an SMS text messaging–based HIV prevention program originally developed in 2013‐2014 for cisgender gay, bisexual, and queer adolescent boys [25-27].

G2G was grounded in the information-motivation-behavioral skills model, which proposes that health behavior change requires not only information about the target behavior but also motivation and skills to engage in the behavior [28-31]. G2G’s active condition was comprised of 5 weeks of 8‐10 daily automated SMS text messaging pushed to adolescents’ phones. Six weeks later, participants were given a week of booster content to reinforce what they had learned. These messages aimed to decrease condomless sex (the primary outcome) and increase HIV prevention knowledge, motivation, self-efficacy, and HIV and sexually transmitted infection (STI) testing behavior (secondary outcomes). The intervention condition also included several interactive elements to promote engagement, including quizzes to test information retention, a question-and-answer feature called G2Genie that enabled participants to request additional texts on a circumscribed number of topics that were ancillary to the primary intervention content (eg, relationships), and badges. Finally, participants were matched with an anonymous “Text Buddy” (another participant in the program) to encourage social support and engagement. G2G’s control condition was an information-only, healthy lifestyle control that did not include interactive components. A pilot randomized controlled trial (RCT) demonstrated G2G’s feasibility, acceptability, and preliminary efficacy, whereby the intervention condition was associated with a 3-fold increase in HIV testing, but no significant difference in condomless sex behaviors compared to the control condition [25-27].

This study was motivated by the question of whether the G2G intervention, if updated and repackaged to focus more on HIV testing, could be effective among a broader group of SGM adolescents who may benefit from HIV testing based on their sexual history. First, we aimed to systematically adapt G2G’s content and delivery to refocus the content on HIV testing as a primary outcome [25-27]. We also sought to reflect significant advancements since the pilot RCT in digital technologies, HIV prevention science, and changes in youth and SGM culture, and to make the intervention content more inclusive of SGM adolescent populations underrepresented in HIV prevention and testing research [32]. Our second aim was to evaluate the intervention’s effectiveness on the new primary outcome of HIV testing and secondary outcomes related to sexual health care engagement, such as PrEP uptake and STI testing, via a nationwide RCT among SGM adolescents. We hypothesized that participants in the intervention condition would be more likely to report HIV testing at least once during the 9-month study period than participants in the control condition. Finally, our third aim engaged experts and adolescents to identify key strategies for reaching and engaging SGM adolescents for digital sexual health interventions in real-world settings. This study is in progress, and of note, after the study launched, various challenges led to modifications of study activities originally proposed in the grant, which will be described in this protocol paper.

## Methods

### Study Overview

**Figure 1.**
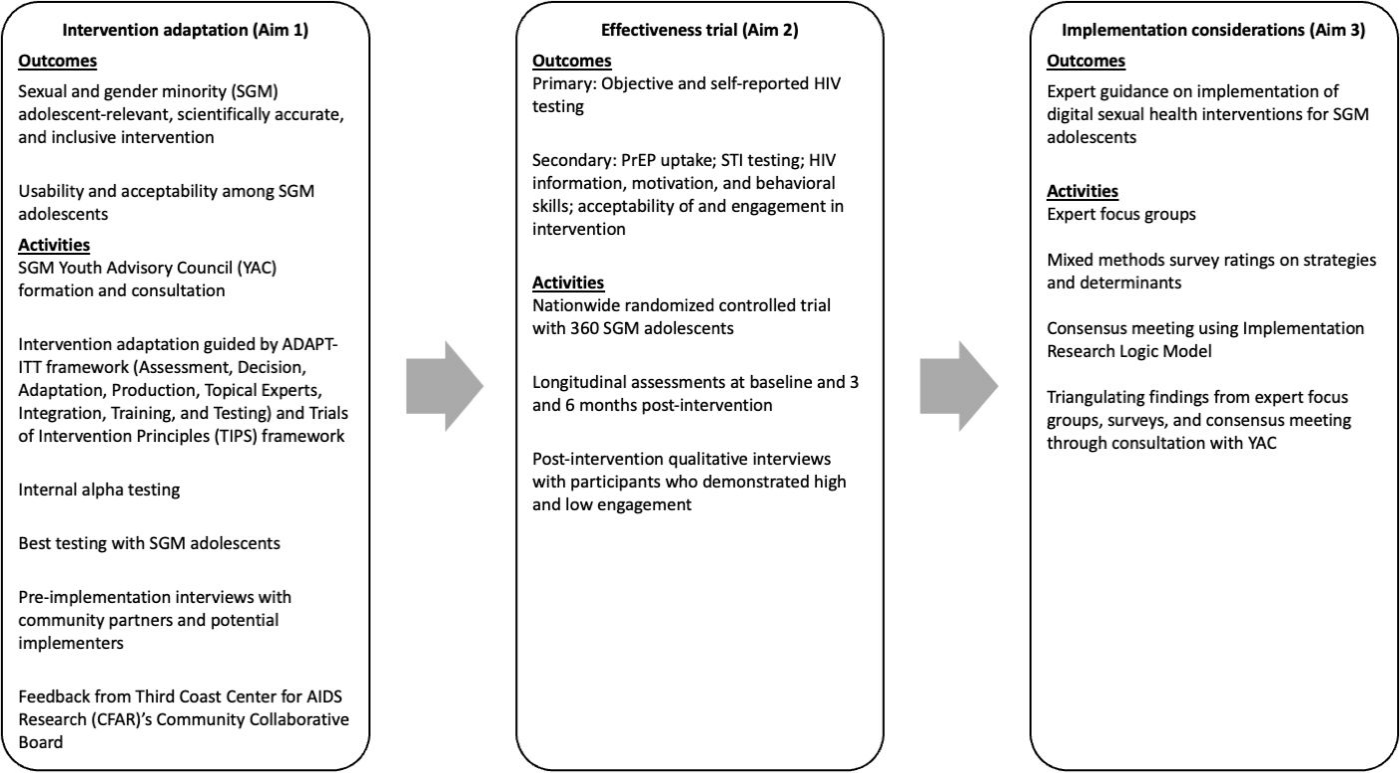
An overview of the study aims with their respective activities and main outcomes. PrEP: pre-exposure prophylaxis; STI: sexually transmitted infection.

This study uses a type 1 hybrid effectiveness-implementation design [33], which primarily tests the intervention’s effectiveness and secondarily explores barriers and facilitators to future implementation. As the study is ongoing, verb tenses in the methods section may be in past or present tense depending on activity completion status. Here we provide an overview of the study aims, and the methods in each aim are described in more detail in the sections below (see Figure 1).

#### Aim 1

From 2022 to 2024 we adapted and modernized G2G content and rebranded the intervention as Sharing Health Education Resources (SHER), which is inclusive of diverse gender identities and reflects HIV testing and prevention innovations since the original G2G RCT. This process was guided by an adolescent advisory council and by pre-implementation interviews with adults who had experience working with SGM adolescents in clinical, educational, and community settings. End user and implementer input was sought to ensure that the intervention content was relevant to adolescents and to increase the likelihood of uptake in real-world settings.

#### Aim 2

From 2024 to 2026 we are conducting a nationwide RCT with SGM adolescents aged 13‐19 years (proposed n=360) to test the effectiveness of SHER compared to an information-only control arm. The primary effectiveness outcomes include objective and self-reported HIV testing, and secondary effectiveness outcomes include sexual health care engagement behaviors, such as PrEP uptake and STI testing. We are enrolling a diverse sample to reflect demographic groups who could benefit from HIV testing yet are underrepresented in adolescent HIV research (eg, Black and Latino youth; transgender and gender-diverse youth; and cisgender girls).

#### Aim 3

In 2025, we gathered feedback from individuals with expertise in reaching and engaging SGM youth in clinical, educational, or community settings; implementing SGM-inclusive sexual health education initiatives; or implementing digital interventions in real-world settings. Through engagement with these experts and SGM adolescents, we identified implementation strategies that effectively address barriers and facilitators to reaching and engaging SGM adolescents for real-world digital interventions and will develop consensus recommendations for dissemination.

### Approach for Aim 1

#### Initial Content and Software Adaptation Phase

##### Content Adaptation

Content revisions followed a modified ADAPT-ITT (assessment, decision, adaptation, production, topical experts, integration, training, and testing) framework [34], a stepwise model for adapting evidence-based HIV interventions. As the first 3 steps of ADAPT-ITT were complete (assess the needs of the target population, decide whether to adopt or adapt interventions, and, if adapting, use a methodology to guide adaptation), we began with the production phase, whereby we created an adaptation plan and determined our adaptation goals. In the following sections of Aim 1 and 2, we allude to other steps of ADAPT-ITT (topical experts to provide content and technical assistance, integrate all forms of information, train personnel, and test for adaptation effectiveness).

First, content from the original G2G program was reviewed by the study team, who consisted of investigators and staff with expertise in SGM adolescent health, HIV prevention, and mental health. The goals were to identify texts that were scientifically outdated, did not reflect contemporary language used by SGM adolescents, or were specific to cisgender boys (eg, references to “guys” and anatomy). Further, reviewers identified possible areas where content focused on HIV testing and other topics desired by youth who completed the G2G pilot trial (eg, relationships and consent) could be expanded. SMS text messages were drafted to address these content areas based on up-to-date scientific knowledge, focusing more on sexual health care engagement outcomes (eg, HIV testing and PrEP) and less on condoms, and were more gender inclusive. To accommodate expanded content and topics such as mental health, communication, and relationships requested by participants from the G2G pilot RCT, we extended the intervention and control arms by 1 additional week. The adapted intervention resulted in roughly 6 weeks of daily SMS text messaging and MMS messages, plus a weeklong booster 6 weeks after this content concluded.

##### Technological Components

The ADAPT-ITT framework was complemented by the Trials of Intervention Principles model [35]. This framework allows intervention developers to assess whether technological or interactive features of a digital intervention can be changed or updated to meet user expectations and preferences without sacrificing the mechanism of behavior change, and enables interventionists to specify how, when, and why technological components are delivered. The study team identified the technical instantiations of the original G2G—SMS text messaging, quizzes, polls, badges, G2Genie, and Text Buddy—and explored which elements were critical to intervention delivery (SMS text messaging) and which elements could be modified to meet modern expectations of technology and youth culture to increase engagement (quizzes, polls, badges, G2Genie, and Text Buddy).

To make quizzes and polls more engaging and meaningful, we developed prototypes whereby participants could earn points that can be exchanged for prizes at the end of the 6-week intervention period; these points replaced the badges in the original G2G. G2Genie enabled participants to text one of several keywords (eg, “relationships”), after which the program would send several SMS text messages about this topic; these texts were static and not updated. To reflect more contemporary online health information-seeking behaviors and technology, we chose to embed links to Planned Parenthood of America’s “Roo,” an artificial intelligence (AI)–powered, evidence-based sexual health chatbot designed for youth that offers dynamic responses to sexual and relationship health questions in real time [36]. Finally, youth in G2G were inconsistently engaged with their Text Buddies [25]; in response, this feature was changed to a group chat where participants are assigned a group of 3‐5 individuals rather than one person to reduce social pressure.

##### Control Condition

**Figure 2.**
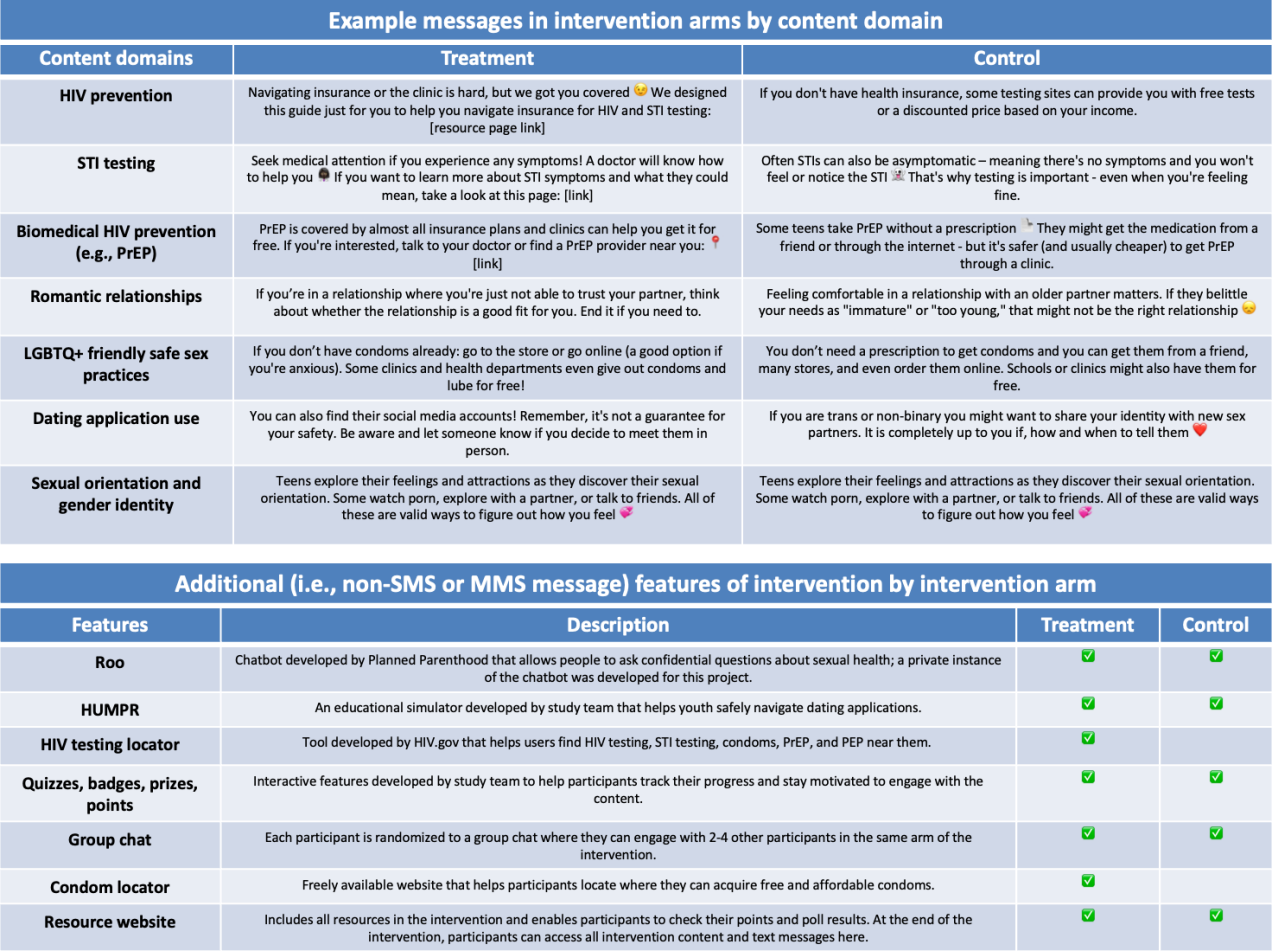
A comparison of SMS text messaging sent in the control and treatment arms and the resources provided in either arm. LGBTQ: lesbian, gay, bisexual, transgender, queer, and questioning; MMS: multimedia messaging service; PEP: postexposure prophylaxis; PrEP: pre-exposure prophylaxis; STI: sexually transmitted infection.

The information-only control group receives the same number of MMS messages and interactive features as the intervention group, over the same amount of time, but does not receive motivational or behavioral skills content. The content, outlined in Figure 2, covers information on physical, social, mental, and sexual health.

### Feedback From Topical Experts

Prototypes of the intervention content and interactive updates underwent review and feedback from a youth advisory council and adult informants as described below.

#### Youth Advisory Council

To ensure SGM adolescent perspectives were reflected in the intervention, we recruited a Youth Advisory Council (YAC) of 24 lesbian, gay, bisexual, transgender, queer, and questioning (LGBTQ+) teens aged 14‐18 (mean 16.6) years. Eligibility criteria included (1) identifying as a sexual and/or gender minority; (2) being 13‐18 years old; (3) reading English at an 8th-grade level or higher; (4) passing a capacity to assent or consent assessment and providing informed consent; and (5) residing in the United States or its territories. YAC members were recruited online via paid social media and online registries. Identity verification procedures were used to confirm eligibility and decrease the likelihood of enrolling impostor participants. This included a video call with a study team member to verify personal information and comprehension of study details [37-39]. Following a virtual orientation session conducted via Zoom (Zoom Communications, Inc), eligible youth were invited to a private Discord (Discord Inc) server, an instant messaging platform that supports MMS, voice, video, and multimedia communication. Members received US $30 per month, with the opportunity to earn an additional US $60 per quarter for optional activities (eg, creating social media content and testing interactive features). For further details on the YAC’s structure and acceptability as a human-centered design approach, see [40].

Each YAC member received components of the adapted intervention in different delivery formats (eg, messages, chatbot, and polls). Members were asked to provide feedback in the Discord server to enhance acceptability, including salience, tone, inclusivity, and engagement with the intervention. Youth also participated in “Wizard of Oz” testing [41], a prototyping approach often used in human-computer interaction and design research, in which study staff manually sent draft versions of messages to YAC members. Participants then gave feedback on the intervention during structured conversations in the Discord server. This enabled the study team to simulate the intervention experience and gather real-time youth feedback on intervention content and features prior to investing considerable time in software development.

#### Interviews With Potential Implementers

To understand potential implementers’ perspectives on the intervention before completing software development and final content edits, we recruited 30 adult participants for 1-hour individual Zoom interviews. Eligibility criteria included being (1) aged 18 years and older, (2) residing in the United States or territories, and (3) working in one of the following areas: adolescent HIV prevention and care (eg, adolescent medicine); digital health; and/or in an organization that uses digital approaches to reach adolescents for services, education, or community programs.

Interviews followed a semistructured guide and were accompanied by a PowerPoint (Microsoft) presentation that outlined the proposed intervention’s target population, intended outcomes, study timeline, sample messages, interactive tools and features (see Figure 2), and proposed implementation models (eg, referral-based vs licensing). Participants were asked for feedback on the content, structure, and delivery of the intervention and to assess its implementation within their organizational settings. Where applicable, depending on the participant’s workplace or experience, additional questions explored strategies for enhancing linkage from digital interventions to HIV and STI and sexual health services. Data were analyzed using qualitative content analysis of the suggested adaptations guided by the Framework for Reporting Adaptations and Modification to Evidence-Based Interventions (FRAME) and FRAME-Implementation Strategies [42,43].

#### Integration of Youth and Adult Feedback

**Figure 3.**
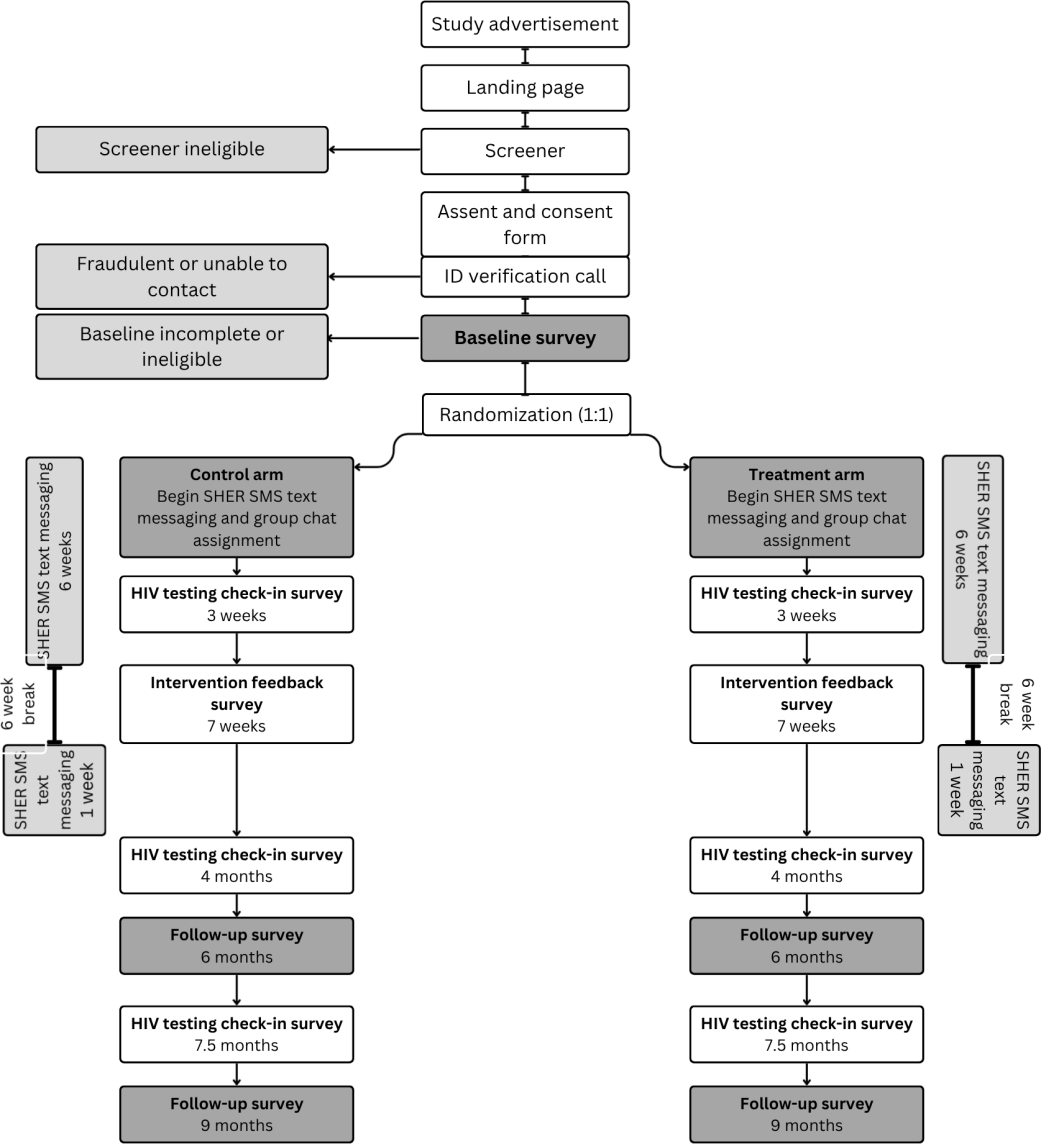
Recruitment, enrollment, and study time points. SHER: Sharing Health Education Resources.

Key themes from the interviews were discussed during internal team meetings, and follow-up questions about intervention content, interactivity, structure, and delivery were posed to the YAC. We focused on areas where perspectives of adolescents and prospective implementers differed, and insights from both groups were used to finalize the content and features of the intervention. The research team finalized intervention content and the delivery of the interactive elements based on our synthesis of this information, and the software development team began finalizing and programming intervention features described in Figures 2 and 3.

### Software Development and Functionality Testing

An in-house research software development team created the bespoke intervention software for the project and is responsible for end-to-end quality assurance testing, troubleshooting, maintenance, optimization, and system hardening (making the system more secure by fixing weaknesses and reducing risks) for the duration of the project. The intervention software and administrative dashboard were developed with study team input that was informed by experience developing prior digital interventions as well as youth and potential implementers’ perspectives. Once content was finalized, functionality testing began. The research team first conducted internal alpha testing (n=14) of both the intervention and control conditions. This phase of testing helped identify bugs, confirm randomization procedures, and ensure functionality of interactive components, allowing the team to engage directly with the MMS delivery platform and make minor adjustments to the intervention content (eg, addressing typos). Minimal errors were identified in this phase.

To streamline data collection, the study team opted to conduct a single round of beta testing, with 3 simultaneous cohorts of testers receiving the intervention over 3 months. A total of 19 adolescent beta testers recruited in the same manner as the YAC members and who reflected the study population participated in this phase, receiving either treatment (n=10) or control (n=9) arm messages over 6 weeks. These beta testers were invited to a private Discord server separate from the YAC to offer feedback on the intervention in real time. Beta testers completed polls, open-ended questions, and prompts specifically designed to evaluate program features and ensure that the technology, such as message delivery, timing, and formatting, was functioning as intended. Bugs were reported in real time through Discord, documented in an Excel file by study staff, and relayed to the software development team for resolution. To further assess functionality and user experience, beta testing concluded with brief phone interviews focused on identifying technical challenges, evaluating the interactive components, and gathering suggestions to enhance engagement. This process confirmed the feasibility and acceptability of the intervention prior to the launch of the RCT in Aim 2.

### Approach for Aim 2

This RCT was initially registered on ClinicalTrials.gov on October 18, 2023 (NCT06096519) and reporting standards from SPIRIT (Standard Protocol Items: Recommendations for Interventional Trials) 2025 were followed [44] (Checklist 1).

### RCT Launch (June 2024-February 2025)

#### Overview

The recruitment, enrollment, and study time points across both arms of the study are outlined in Figure 3. The RCT was launched in June 2024, and initial eligibility criteria were (1) male gender identity or male sex at birth, (2) ever had anal sex with a partner assigned male at birth, (3) identify as a sexual or gender minority or are questioning their sexuality, (4) aged 13‐18 years, (5) read in English at a 6th grade level, (6) never received an HIV or STI test, (7) HIV negative or unknown status, (8) own a cell phone with an unlimited MMS plan and plan to have the same number during the study, (9) can provide informed consent, as shown on a capacity to consent assessment [45], and (10) live in the United States or US territories. Like the YAC, participants initially were recruited predominantly via social media platforms, where they viewed advertisements that directed them to a study information page, followed by a brief online screener and a required identity verification call. Informed consent and assent are completed online by the participant, followed by a video chat with study staff to answer questions and/or address concerns about the study prior to enrollment. All personal information from potential and enrolled participants is collected remotely and stored in REDCap (Research Electronic Data Capture; Vanderbilt University) and private university servers.

#### Randomization

After screening for eligibility, verifying identity, and completing the baseline survey (described in Assessment below), enrolled participants were randomly assigned to receive SHER or the information-only control arm (1:1 allocation). Randomization is stratified by age (minors vs 18- and 19-year-olds), sex assigned at birth, prior condomless penetrative sex with a partner assigned male at birth, and gender identity at baseline to ensure sufficient representation by these characteristics in each condition to conduct exploratory analyses of potential group differences in outcome effects. After incorporating changes to eligibility criteria described below, HIV testing history (never or ever received an HIV test) was added as a stratification variable to account for the inclusion of individuals that have previously been tested. A computerized minimization technique [46-49] is implemented to reduce the likelihood of imbalance across study arms with respect to our stratification variables. Personnel who enroll the participants do not have access to the random allocation sequence. Participants are blinded and are only told they are testing one of 2 versions. Participants will not be unblinded until after all study data are collected.

#### Recruitment Challenges (July 2024-August 2024)

Initial recruitment was relatively slow and costly, with only 24 participants enrolled over a 5-month period at a cost of US $273.05 per participant. This cost was over 4 times the cost per enrollment (US $61.22) using the same Meta advertising strategy as a similar digital sexual health clinical trial reaching a nearly identical population in years 2018‐2020 [50,51]. The study team hypothesized that this was largely due to policy changes implemented by Meta in 2023 that increased restrictions on targeted advertisements to minor adolescent users (eg, prohibited from targeting minors based on sexual or romantic interest in same-sex partners) [52].

In July and August 2024, the research team observed a surge in eligible screener entries. However, after nearly all these individuals failed identity verification calls for similar reasons, it became clear that they were likely “impostor participants” [53]. Given that the new group chat feature enabled adolescent participants to interact with other participants, additional procedures were implemented to protect participant safety and data integrity. A modification was submitted to the institutional review board (IRB) in August 2024 to update the screening and verification procedures with more robust methods, such as requiring photo identification (eg, school ID or government ID), additional verification questions, and careful screening of photo IDs for AI generation and editing. During the one-month period of IRB submission and review, online study advertising was paused and resumed at the end of August 2024. These procedures, though time-consuming, appeared to act as an effective deterrent.

#### Changes to Inclusion Criteria (March 2025)

After several months of recruitment, it became clear that the recruitment procedures and inclusion criteria that were used in the study team’s prior intervention research with SGM adolescents were not yielding a robust sample [51]. The study team attempted to reduce bottlenecks to enrollment by reducing the number of steps from advertisement click to screener completion, reducing the number of screener questions, and making the study information page more engaging. However, the most substantial obstacle to enrollment appeared to be the stringent inclusion criteria. Following consultation with the investigative team and the study’s program officer at the US National Institutes of Health, several changes were made.

First, criteria were expanded to include participants that had ever had oral sex, but not penetrative sex, with a partner assigned male at birth. Our screening data showed that 404 individuals had been excluded from participation based on sexual experience alone. Evidence suggests most adolescents initiate oral and penetrative sex within the same 6-month period [54]. Therefore, individuals with only oral sex experience were presumed likely to engage in behaviors that would warrant HIV testing during the 9-month study period, as shown in the survey timeline depicted in Figure 3. In addition, 174 individuals had been excluded during initial recruitment between July 2024 and February 2025 based on prior experience with HIV and STI testing. We thus chose to include individuals who had previously been tested, were not taking PrEP (as PrEP regimens require roughly quarterly HIV testing), and could benefit from HIV testing based on clinical guidelines.

Next, age criteria were expanded to include 19-year-olds, who are still in the developmental stage of adolescence yet are more likely to be sexually experienced and still report low HIV and STI testing rates [55]. Thirteen individuals were excluded during the initial recruitment period based on being 19 years of age. Finally, in June 2025, we expanded the intervention to include cisgender sexual minority (eg, lesbian, bisexual, pansexual, and queer) girls who reported vaginal or anal sex with a partner assigned male at birth. Expanding criteria to be inclusive of more gender identities was favored by community partners and prospective implementers, as this would serve a greater proportion of their client population and reach a group who could benefit from but may not be targeted for HIV and STI screening [56].

Before implementing these expanded inclusion criteria, the study team audited and adapted the intervention content to ensure relevance and appropriateness for the newly included demographics. Enrollment rapidly increased after relaunching the study with updated criteria in March 2025, and cost per enrolled participant was reduced to US $70.61 as of September 2025, which is consistent with the team’s previous work with SGM adolescents in digital health studies [51].

#### Changes to Sample Size (August 2025-March 2026)

In summer 2025, although the enrollment rate increased, it was not enough to compensate for the time lost in the earlier months of the RCT, and the team was experiencing several additional challenges. First, there was uncertainty about whether the project would continue to be funded by NIH due to changes in federal funding priorities, and the grant budget had been reduced substantially. Furthermore, RCT and Aim 3 activities were occurring simultaneously due to the recruitment delays, requiring study staff to manage competing priorities. In addition, the longer recruitment lasted, the more costs would be incurred for advertising and retention. These factors led the investigative team to explore the feasibility of reducing the sample size to conserve resources and ensure completion of the trial before the end of the grant period.

Power calculations were performed using nominal type I error rates (α=.05) and assumed a 1:1 allocation to each study arm and identical arm-specific retention rates. The assumed HIV testing rate for control participants at the final time point was 28% based on previous findings from the G2G pilot study [26]. The original sample size proposed in the grant (n=360) was chosen to power comparisons at the end of the trial. Assuming 80% retention, the study would be 81% powered to detect a small to medium intervention effect for self-reported or objective HIV testing. Revised power calculations were conducted in August 2025 by a biostatistician. It was concluded that, assuming study endpoint retention rates of 90% or higher, the study’s target sample size could be reduced from 360 to 310 and maintain the 80% power to detect differences between the intervention and control groups at the same magnitude that was targeted in the original power analysis. At the time of these analyses, study retention was at or near 90% and, in March 2026, exceeded 90%. The sample size reduction and expanded inclusion criteria will likely limit the ability to conduct adequately powered subgroup analyses, so those analyses will be treated as exploratory. However, maintaining the higher targeted retention rate should allow for mediation analysis at similar power levels as the originally targeted sample size. Assuming these recruitment rates hold, we anticipate completing recruitment by June 2026 and retention efforts by January 2027.

During the October 2025 Data Safety Monitoring Board (DSMB) meeting, we informed board members about our plan to reduce the sample size. The DSMB unanimously agreed this was the appropriate course of action, noting our high retention rates exceeding 90% and the prolonging of recruitment to obtain our original sample size was unnecessary. The principal investigator also sought approval from the NIH in October 2025, which was granted in March 2026, following the submission of our annual research performance progress report.

#### Pre- and Postlaunch Protocol Modifications

In summary, our study originally aimed to enroll 360 participants between the ages of 13 and 18 years who were assigned male at birth or had a masculine gender identity, had not previously obtained an HIV test, and had engaged in penetrative sex with a partner assigned male at birth. In March 2025, we expanded the criteria to include 19-year-olds, individuals assigned female at birth who were at elevated risk for HIV, and those who had previously obtained an HIV test but at the time of enrollment were eligible for another HIV test. Despite implementation of multiple recruitment strategies, recruitment continued to progress slower than anticipated. In 2026, the study team therefore reduced the sample size to 310, having determined that statistical power would be retained provided retention rates remained at or above 90%, which was highly likely based on interim retention rates. All modifications were approved by the IRB and NIH prior to implementation, and ClinicalTrials.gov was updated accordingly.

#### Assessment

Measures (Table 1) are collected at baseline, immediately postintervention, and 3- and 6-month postintervention. Baseline measures assess HIV and STI testing behaviors, HIV-related knowledge, motivation, and behavioral skills, sociodemographic characteristics, and sexual history. All participants complete an assessment immediately postintervention (6-week) to evaluate intervention acceptability adapted from a previous digital sexual health study [50,57]. At 3-month postintervention, HIV information, motivation, and behavioral skills [58] and HIV and STI testing history are assessed again. Then, we follow adolescents for 6 additional months (Table 1) to allow enough time to elapse for participants to engage in HIV testing. The final 6-month postintervention survey assesses the same constructs as the 3-month survey. Finally, qualitative interviews with participants from treatment and control arms (n=20 each), who demonstrate low and high engagement in SHER (n=20 each) will further assess acceptability and completion barriers. Level of engagement is defined by the number of prompts (ie, quizzes and polls) that participants responded to during the intervention (low engagers ≤9 responses, high engagers >9 responses).

**Table 1.** Randomized controlled trial (RCT) outcome measures: operationalization and schedule.

Constructs	Measures/operationalization	Measurement timeline						
		Baseline	3 weeks	6 weeks	3 months	6 months	7.5 months	9 months
HIV testing (primary)	Self-reported HIV testing or objective proof of HIV testing in the previous 3 months [51]	✓	✓		✓	✓	✓	✓
STI^a^ testing (secondary)	Self-reported history of testing for STI in the previous 3 months [51]	✓	✓		✓	✓	✓	✓
PrEP^b^ (secondary)	Self-reported at the time of the baseline assessment and past 3-month PrEP use, and willingness to use PrEP [59,60]	✓				✓		✓
Sexual experience history (secondary)	Lifetime and past 3 month sexual experiences, including condomless anal sex partners [61]	✓				✓		✓
Condom use, intentions, and self-efficacy (O)^c^	Condom Use Intentions Scale–11 items; Condom Use Self-Efficacy Scale–5 items; Adapted condom errors questionnaire–15 items [62-64]	✓				✓		✓
HIV knowledge (mediator)	Knowledge of HIV transmission and prevention [65]	✓				✓		✓
Motivation and behavioral skills (mediator)	Motivation (eg, motivation to improve sexual practices), self-efficacy to access HIV testing and prevention methods, social norms (eg, partners', friends’, or family members’ opinions about condom use), and behavioral skills (eg, negotiating condom use) [63]	✓				✓		✓
Behavioral health (O)	Self-reported substance use history, perceived social support, internalized stigma, anxiety, depression, and stress [50,51,66]	✓				✓		✓
SHER^d^ intervention acceptability and tolerability (O)	Intervention impact, usefulness, engagement, usability [50,51]			✓				
Demographics (moderator)	Self-reported age, gender identity, sexual orientation, race and ethnicity, education, socioeconomic status, and rural versus urban [67]	✓						
Dose and engagement (moderator)	System-captured data on intervention dose and engagement (eg, number of texts sent to buddy, number of links clicked, and number of times chatbot used)			✓				

a STI: sexually transmitted infection.

b PrEP: pre-exposure prophylaxis.

c O: other measure.

d SHER: Sharing Health Education Resources.

### Outcome Measures

Our primary effectiveness outcome of HIV testing is measured using self-report and objective proof (eg, screenshot or photo of HIV testing results uploaded to REDCap, our survey software) while secondary effectiveness outcomes (eg, PrEP uptake and STI testing) are measured via self-report only. We remind all adolescents across study arms at multiple time points (consent, ID verification call, and active or control arm messages related to HIV testing, and boosters) to retain evidence of testing. As some may not notify us immediately, each follow-up survey asks for testing experiences since the prior assessment. Those who report being tested are prompted to securely upload evidence of their HIV or STI test via a participant-specific REDCap link in line with procedures from prior work [68,69]. If any participants report testing positive for HIV or STIs, a staff member will promptly contact the participant and link them to care.

#### Quantitative Analysis

We hypothesize that participants in the SHER intervention and control groups will differ in whether they are tested for HIV during the study and that HIV prevention, testing, and treatment information, motivation, and behavioral skills will mediate these effects (Table 1). Our secondary outcomes will include PrEP initiation; discussion with providers about HIV, STI testing, and PrEP; and frequency of receptive or insertive condomless sex and STI testing.

We will assess the experimental and control groups’ similarities at baseline. Variables that differ significantly will be covariates in adjusted outcome analyses, along with variables correlated with the outcome (eg, age), as well as covariates associated with missingness in order to address differences in attrition [70-74]. The primary outcome will be analyzed using a generalized linear mixed effects model that assumes a binomial distribution and logit link. The generalized linear mixed effects model will incorporate data from baseline, 3-month, and 6-month follow-ups and account for both fixed and random effects over time. Residual diagnostics will be used to evaluate model fit, including the possibility of overdispersion. We will report study arm coefficients as log odds ratios and report odds ratio point estimates, SEs, and 95% CIs. The test of the treatment effect will be 2-sided Wald tests that control the type I error rate at α=.05. Missingness will be addressed using either full information maximum likelihood (if missingness is less than 25% as anticipated) or multiple imputation (if missingness is greater than 25%) [75].

#### Qualitative Analysis

Postintervention interview transcripts will be uploaded to Dedoose [76,77]. Directed content analysis will identify and summarize intervention acceptability and completion barriers or facilitators [78-81]. Codes will be iteratively refined and applied to the data until reliability reaches kappa 80% [82].

#### Data Security and Participant Safety

A DSMB composed of 3 independent experts was assembled to ensure participant safety, data integrity, and ethical conduct. The first meeting occurred in April 2024 prior to the start of the trial. The board meets every 6 months, with the final meeting planned for after data collection concludes. During meetings there is (1) an open session in which the study team reviews the progress of the study and answers questions from members of the DSMB; (2) a closed session involving DSMB members (if determined necessary) where the study statistician discusses quantitative outcomes and answers questions; and, if requested, (3) a final session involving DSMB members only to discuss study progress and outcome results, develop recommendations, and take votes as necessary. Each meeting is led by a chair, which alternates every meeting and is responsible for issuing a final recommendation to continue, modify, or end the study.

Participant data will be coded with randomly generated identifier codes; responses will never be linked to a participant’s name, email address, phone number, or any other identification information. All data presented will be done in aggregate without any personal identifiers. All survey data will be stored in REDCap and housed on Northwestern University computers and servers. Participants in interview portions of the study participate in a Zoom interview or telephone interview if they are unable to use Zoom. Audio is recorded via Zoom or digital audio recorder and transferred to secure Northwestern servers immediately after the interview. Following this, audio files are deleted from local devices. Interview audio is uploaded to a secure online transcription service. All interview data are deidentified by research staff prior to entry into a cloud-based qualitative data analysis software. Deidentified data are stored indefinitely on secure shared drives on university servers.

During the trial, participants provide their phone numbers and receive automated SMS text messaging and MMS messages from a dedicated Northwestern server that delivers the intervention. Paradata such as engagement with SMS text messaging (eg, replying to question prompts sent via text) is transmitted back to university servers and exported into Excel (Microsoft) files for analysis. Group SMS text messages are routed through the server and delivered to participants. This avoids participants having to disclose phone numbers with others in their group, enables recording of SMS text messaging for analysis, and allows the study team to monitor group texts for inappropriate or bullying behavior.

### Approach for Aim 3

Aim 3 activities were focused on identifying expert- and adolescent-recommended strategies for reaching and engaging SGM youth in digital sexual health interventions. Specifically, this aim identified, specified, and prioritized effective strategies for increasing a digital health intervention’s reach, defined as adolescents who are aware of the digital intervention and decide to participate, and engagement*,* defined as adolescents who continue to use or participate in the digital intervention. The overarching goal was to provide any individual or organization who aims to include SGM adolescents in digital outreach, programming, or interventions (eg, researchers, industry partners, and community organizations) with expert guidance that can enhance their ability to reach and engage these adolescents.

To achieve this goal, concurrent with Aim 2 activities, we recruited 18 individuals in the United States who had expertise in SGM health, adolescent health, HIV prevention, sexual health, digital health interventions, digital marketing, or other areas relevant to SHER. Experts were recruited via professional networks, snowball referrals, and cold-emailing different organizations identified through social media and internet-based searches. This purposive sampling approach was taken to ensure that the panelists reflected the appropriate areas of expertise to offer comprehensive and trustworthy recommendations for reaching and engaging SGM adolescents in a variety of settings.

This aim used a pragmatic sequential mixed methods process modeled after the methods used to develop the Expert Recommendations for Implementing Change, a taxonomy of expert-identified implementation strategies [83,84]. The process consisted of 3 phases, including identifying strategies to reach adolescents and determinants of these strategies, rating these strategies and determinants to understand how experts prioritize them, and connecting and building upon findings from the first 2 phases via discussion and consensus. Experts were compensated US $1000 for participating in several activities over approximately 6 months, including 2 surveys, a focus group, and a 4-hour-long panel meeting.

### Identifying Determinants and Implementation Strategies for Reaching and Engaging Adolescents

This phase consisted of a series of 90-minute focus groups conducted via Zoom [85] with 3‐5 expert panel members each in April-May 2025. Focus groups were recorded and transcribed. Prior to each focus group, each expert completed a brief demographic survey that included 3 open-ended questions, “What are some of the most successful ways you have reached and engaged SGM teens in your programs? These could be different ways for reaching teens vs engaging teens in programs” “What has gotten in the way of successfully reaching and engaging SGM teens in your program?” and “What has made it easier to successfully reach and engage SGM teens in your program*?*” These survey responses were used by the research team to identify common and unique themes across panelists to guide the focus group discussion.

During the focus groups, experts were asked to discuss these practices (ie, strategies) in their respective organizations for reaching and engaging SGM adolescents. The research team prompted panelists to specify the actors who do the strategy, the specific actions taken, the temporality of when the strategy is used, and the dosage (frequency and time) of each strategy [86]. This prompting focused on identifying the rationale or justification for using a specific strategy, with a focus on identifying specific barriers and facilitators to reaching and engaging SGM adolescents that would be addressed by the strategy. The research team then engaged in rapid qualitative content analysis of the focus group transcripts and facilitator notes to generate a preliminary list of strategies corresponding to barriers and facilitators [78-81].

### Rating Reach and Engagement Strategies and Determinants to Inform Prioritization

A comprehensive list of all reach and engagement strategies, barriers, and facilitators identified during the focus groups was prepared by the research team following consolidation of conceptually similar items. These lists were presented to the experts through an online survey sent in July 2025, which asked them to rate both the feasibility and importance of each strategy using a 10-point Likert scale (1: least-10: most). Determinants were assigned as a barrier or facilitator based on information and context from the focus groups. Panelists were asked to rate each determinant on a 3-point scale based on their respective influence on reach and engagement of SGM adolescents ([1] small impact, [2] medium impact, and [3] strong impact) [87]. Open-ended questions for reach and engagement strategies and determinants enabled experts to provide additional context for their ratings. Descriptive statistics were conducted to summarize the average feasibility and importance of each strategy and the relative impact of the barriers and facilitators. The open-ended responses were analyzed using rapid qualitative content analysis [78-81]. The research team used the survey data to generate the strategies and determinants sections of an implementation research logic model, which indicated the relationships between determinants and strategies [88-90].

### Building Consensus on Reach and Engagement Strategies and Determinants

The final phase included a half-day online consensus meeting in September 2025 with the expert panelists to finalize the list of best practices for reaching and engaging SGM adolescents in real-world digital health interventions. The list of best practices was presented to the panelists to facilitate discussion focused on resolving discrepancies that emerged from the focus group and survey results, achieving expert consensus on strategies to reach and engage SGM adolescents, and identifying additional considerations for real-world delivery [83,84]. The meeting also included a discussion of recommended dissemination strategies to inform the development of a strategy toolkit. In October 2025, the conclusions from the consensus meeting with experts were reviewed by the YAC, who offered additional recommendations and considerations about what strategies they perceive are best for reaching and engaging youth like them. In December 2025, the study team began developing a digital toolkit focused on sharing expert-identified strategies for reaching and engaging SGM youth, which will be disseminated to both academic and nonacademic audiences seeking to work more effectively with this population.

### Ethical Considerations

The study was approved on August 27, 2022, by the Northwestern University IRB (IRB00217358) and was determined to be minimal risk in nature. Guardian permission was waived for minor adolescent participants. All participants provided informed consent via an online consent form in REDCap prior to engaging in study procedures. Participant compensation for different stages of the study is described in the Results section. Data are deidentified and stored in secure university servers only accessible to IRB-approved study team members. Modifications to the study protocol were submitted to the IRB for approval when applicable.

## Results

The study was funded in April 2022 by the National Institute of Mental Health (R01MH129207, principal investigator: KM). Participants in the Aim 1 YAC were recruited beginning in January 2023. Recruitment for the council continued on a rolling basis to replace participants that withdrew or aged out until the conclusion of data collection in January 2025. Aim 1 stakeholder recruitment also began in January 2023, with data collection concluding in October 2023. Results of Aim 1 are expected to be published in Fall 2026. Aim 2 RCT recruitment began in June 2024. As of April 2026, 296 participants have been enrolled. Data analysis and publication of results of Aim 2 activities are expected to occur in Winter 2027. The Aim 3 expert panel was recruited in March and April of 2025, with all data collection activities associated with this aim concluding in September 2025. The results of Aim 3 are expected to be published in Summer 2026.

## Discussion

### Principal Findings

Digital interventions that respond to SGM adolescents’ HIV prevention and testing needs and are tailored to their lived experiences remain scarce. In this study, we modernized and adapted an efficacious intervention (G2G) that we are testing in an effectiveness-implementation trial. Further, it is estimated to take roughly 17 years for only a fraction of evidence-based interventions to reach routine care [91]. Thus, we aimed to address this research-to-practice gap by working with experts and adolescents to identify strategies that will reliably reach and engage SGM adolescents into evidence-based digital interventions as well as other types of programs for these youth.

Intervention and technology refinements were informed by iterative feedback from youth, potential implementers from clinical, community, digital health, and educational settings, and public health experts to ensure acceptability and implementation potential. We retained the core information-motivation-behavioral model of G2G while modernizing content, increasing inclusivity, and embedding novel interactive components such as group chats and an AI-powered chatbot developed by a trusted national sexual and reproductive health care organization [92]. These changes align with adolescent preferences for participatory, conversational, and affirming digital health tools [93,94]. SHER’s integration of these interactive features represents a meaningful evolution of SMS text messaging–only HIV prevention and testing interventions, making it better suited to modern expectations for digital engagement and peer connectivity.

Initial recruitment challenges prompted mid-trial revisions to our eligibility criteria, which made the study open to a much broader group of SGM adolescents. These changes were necessary due to a combination of newer constraints in digital advertising to minors, increases in fraudulent participants in online research, and high ineligibility rates. Despite these challenges, the expansion of inclusion criteria arguably makes the study more pragmatic than a typical hybrid type-1 study and can enable us to assess SHER’s relevance for youth who are often excluded from HIV prevention and testing intervention research [95,96]. These adaptations were consistent with similar protocol revisions in other digital HIV prevention trials affected by contextual shifts in digital marketing and sexual health behaviors during and after the COVID-19 pandemic [97]. Our experiences suggest that digital HIV prevention efforts for adolescents must contend with shifting sociotechnical landscapes, new challenges that threaten data validity and participant safety (eg, AI-generated responses, bots, and imposter participants), changes in youth culture, advances in HIV prevention technologies, and evolving digital privacy policies.

Importantly, this study also addresses the “implementation gap” [91] by incorporating an aim that centers on participatory expert consensus building to identify strategies for reaching and engaging SGM adolescents in the real world. This approach is informed by models such as Expert Recommendations for Implementing Change and the Consolidated Framework for Implementation Research (CFIR) [87] and complements our RCT findings with actionable insights for real-world delivery. By involving both implementation experts and SGM adolescents, we ensure that resulting guidance reflects both strategic and experiential knowledge.

There are limitations to our design. Perhaps most notably, our expansion of inclusion criteria to ensure trial completion, combined with our time and budget constraints, may preclude us from examining SHER’s effectiveness on subgroups of SGM adolescents for whom the original G2G intervention was not designed. Further, our study relies on self-reported outcomes, which may introduce social desirability bias, though our primary outcome is supplemented by objective evidence of HIV testing to address this issue. Additionally, the intervention has multiple components aimed at increasing engagement, but given our trial design, it is unclear which, if any, of these has a meaningful impact on the outcome of HIV testing. Exploratory dose response analyses will help clarify which components of the intervention are most effective and whether higher engagement predicts stronger outcomes. Future studies may consider other pragmatic trial designs (eg, multiphase optimization strategy [98]) that can disentangle which interactive components are most critical to supporting behavior change.

### Conclusion

This study seeks to address multiple gaps in adolescent HIV prevention in the United States by expanding and testing an efficacious digital health intervention aimed at promoting knowledge, motivation, and skills to engage in HIV and STI testing behaviors among SGM adolescents engaging in, or likely to engage in, HIV transmission risk behavior. Further, the study engages adolescents and adults with expertise in working with SGM adolescents throughout to inform the intervention and its eventual implementation, with a specific focus on identifying strategies to reach adolescents who are historically challenging to engage in, and thus excluded from, prevention efforts.

## Supplementary material

10.2196/88465 Checklist 1 SPIRIT checklist.

10.2196/88465 Peer Review Report 1 Peer review report by: HIBI - HIV/AIDS Intra- and Inter-personal Determinants and Behavioral Interventions Study Section, Risk, Prevention and Health Behavior Integrated Review Group, National Institute of Mental Health (National Institutes of Health).

## Abbreviations

ADAPT-ITT: assessment, decision, adaptation, production, topical experts, integration, training, and testing
AI: artificial intelligence
CFIR: Consolidated Framework for Implementation Research
DSMB: Data Safety Monitoring Board
FRAME: Framework for Reporting Adaptations and Modification to Evidence
G2G: guy2guy
IRB: institutional review board
LGBTQ+: lesbian, gay, bisexual, transgender, queer, and questioning
MMS: multimedia messaging service
PrEP: pre-exposure prophylaxis
RCT: randomized controlled trial
REDCap: Research Electronic Data Capture
SGM: sexual and gender minority
SHER: Sharing Health Education Resources
SPIRIT: Standard Protocol Items: Recommendations for Interventional Trials
STI: sexually transmitted infection
YAC: Youth Advisory Council

## References

[1] (2023). Diagnoses of HIV infection in the united states and dependent areas, 2021. https://stacks.cdc.gov/view/cdc/149071.

[2] (2018). Estimated HIV incidence and prevalence in the united states, 2010-2015. Centers for disease control and prevention.

[3] Kann L, McManus T, Harris WA (2018). Youth risk behavior surveillance - United States, 2017. MMWR Surveill Summ.

[4] Wyckoff AS (2023). 2023 periodicity schedule updates HIV screening guidance. American Association of Pediatrics.

[5] (2025). Clinical testing guidance for HIV. HIV Nexus: CDC Resources for Clinicians.

[6] Final recommendation statement: human immunodeficiency virus (HIV) infection: screening. United States Preventive Services Taskforce.

[7] Sharma A, Wang LY, Dunville R, Valencia RK, Rosenberg ES, Sullivan PS (2017). HIV and sexually transmitted disease testing behavior among adolescent sexual minority males: analysis of pooled youth risk behavior survey data, 2005-2013. LGBT Health.

[8] Sharma A, Kahle E, Todd K, Peitzmeier S, Stephenson R (2019). Variations in testing for HIV and other sexually transmitted infections across gender identity among transgender youth. Transgend Health.

[9] Makrides J, Matson P, Arrington-Sanders R, Trent M, Marcell AV (2023). Disparities in sexually transmitted infection/HIV testing, contraception, and emergency contraception care among adolescent sexual minority women who are racial/ethnic minorities. J Adolesc Health.

[10] (2024). Compendium of evidence-based interventions and best practices for HIV prevention: background, methods, and criteria. Centers for Disease Control Division of HIV Prevention.

[11] Harper GW (2007). Sex isn’t that simple: culture and context in HIV prevention interventions for gay and bisexual male adolescents. Am Psychol.

[12] Herbst JH, Sherba RT, Crepaz N (2005). A meta-analytic review of HIV behavioral interventions for reducing sexual risk behavior of men who have sex with men. J Acquir Immune Defic Syndr.

[13] Mustanski BS, Newcomb ME, Du Bois SN, Garcia SC, Grov C (2011). HIV in young men who have sex with men: a review of epidemiology, risk and protective factors, and interventions. J Sex Res.

[14] Faverio M, Teens SO (2024). Social media and technology 2024. Pew Research Center.

[15] Arya M, Kumar D, Patel S, Street RL, Giordano TP, Viswanath K (2014). Mitigating HIV health disparities: the promise of mobile health for a patient-initiated solution. Am J Public Health.

[16] Boulos MNK, Wheeler S, Tavares C, Jones R (2011). How smartphones are changing the face of mobile and participatory healthcare: an overview, with example from eCAALYX. Biomed Eng Online.

[17] Fox S, Duggan M (2012). Mobile health 2012. Pew Res Cent.

[18] Mark T (2010). Medical apps for smartphones. Telemedicine and E-Health.

[19] Ybarra ML, Bull SS (2007). Current trends in Internet- and cell phone-based HIV prevention and intervention programs. Curr HIV/AIDS Rep.

[20] L’Engle KL, Mangone ER, Parcesepe AM, Agarwal S, Ippoliti NB (2016). Mobile phone interventions for adolescent sexual and reproductive health: a systematic review. Pediatrics.

[21] Gelles-Watnick MER, Teens N (2022). Social media and technology 2022. Pew Res Cent.

[22] Widman L, Nesi J, Kamke K, Choukas-Bradley S, Stewart JL (2018). Technology-based interventions to reduce sexually transmitted infections and unintended pregnancy among youth. J Adolesc Health.

[23] Li DH, Brown CH, Gallo C (2019). Design considerations for implementing eHealth behavioral interventions for HIV prevention in evolving sociotechnical landscapes. Curr HIV/AIDS Rep.

[24] Mohr DC, Lyon AR, Lattie EG, Reddy M, Schueller SM (2017). Accelerating digital mental health research from early design and creation to successful implementation and sustainment. J Med Internet Res.

[25] Ybarra ML, Prescott TL, Phillips GL, Bull SS, Parsons JT, Mustanski B (2017). Pilot RCT results of an mHealth HIV prevention program for sexual minority male adolescents. Pediatrics.

[26] Ybarra ML, Liu W, Prescott TL, Phillips G, Mustanski B (2018). The effect of a text messaging based HIV prevention program on sexual minority male youths: a national evaluation of information, motivation and behavioral skills in a randomized controlled trial of Guy2Guy. AIDS Behav.

[27] Ybarra ML, Prescott T, Mustanski B, Parsons J, Bull SS (2019). Feasibility, acceptability, and process indicators for Guy2Guy, an mHealth HIV prevention program for sexual minority adolescent boys. J Adolesc Health.

[28] Fisher CM (2012). Adapting the information-motivation-behavioral skills model: predicting HIV-related sexual risk among sexual minority youth. Health Educ Behav.

[29] Fisher JD, Fisher WA, Williams SS, Malloy TE (1994). Empirical tests of an information-motivation-behavioral skills model of AIDS-preventive behavior with gay men and heterosexual university students. Health Psychol.

[30] Fisher TD, Davis CM, Yarber WL (2013). Handbook of Sexuality-Related Measures.

[31] Fisher WA, Williams SS, Fisher JD, Malloy TE (1999). Understanding AIDS risk behavior among sexually active urban adolescents: an empirical test of the information–motivation–behavioral skills model. AIDS Behav.

[32] Barr E, Marshall LJ, Collins LF (2024). Centring the health of women across the HIV research continuum. Lancet HIV.

[33] Curran GM, Landes SJ, McBain SA (2022). Reflections on 10 years of effectiveness-implementation hybrid studies. FrontHealth Serv.

[34] Wingood GM, DiClemente RJ (2008). The ADAPT-ITT model: a novel method of adapting evidence-based HIV Interventions. J Acquir Immune Defic Syndr.

[35] Mohr DC, Schueller SM, Riley WT (2015). Trials of intervention principles: evaluation methods for evolving behavioral intervention technologies. J Med Internet Res.

[36] (2025). Ask roo | the sexual health chatbot from planned parenthood. Planned Parenthood.

[37] Bauermeister JA, Pingel E, Zimmerman M, Couper M, Carballo-Diéguez A, Strecher VJ (2012). Data Quality in HIV/AIDS Web-Based Surveys: Handling Invalid and Suspicious Data. Field methods.

[38] Grey JA, Konstan J, Iantaffi A, Wilkerson JM, Galos D, Rosser BRS (2015). An updated protocol to detect invalid entries in an online survey of men who have sex with men (MSM): how do valid and invalid submissions compare?. AIDS Behav.

[39] Teitcher JEF, Bockting WO, Bauermeister JA, Hoefer CJ, Miner MH, Klitzman RL (2015). Detecting, preventing, and responding to “fraudsters” in internet research: ethics and tradeoffs. J Law Med Ethics.

[40] Gordon J, Lorenzo J, Avila AA (2025). Engaging LGBTQ+ youth in human-centered design of a digital health intervention via discord: implementation case study (preprint). JMIR Form Res.

[41] Dow S, MacIntyre B, Lee J, Oezbek C, Bolter JD, Gandy M (2005). Wizard of Oz support throughout an iterative design process. IEEE Pervasive Comput.

[42] Miller CJ, Barnett ML, Baumann AA, Gutner CA, Wiltsey-Stirman S (2021). The FRAME-IS: a framework for documenting modifications to implementation strategies in healthcare. Implement Sci.

[43] Wiltsey Stirman S, Baumann AA, Miller CJ (2019). The FRAME: an expanded framework for reporting adaptations and modifications to evidence-based interventions. Implement Sci.

[44] Chan AW, Boutron I, Hopewell S (2025). SPIRIT 2025 statement: updated guideline for protocols of randomized trials. JAMA.

[45] (2003). Procedures for Determination of Decisional Capacity in Persons Participating in Research Protocols. UCSD task force on decisional capacity.

[46] McEntegart DJ (2003). The pursuit of balance using stratified and dynamic randomization techniques: an overview. Drug Information J.

[47] Scott NW, McPherson GC, Ramsay CR, Campbell MK (2002). The method of minimization for allocation to clinical trials. a review. Control Clin Trials.

[48] Taves DR (1974). Minimization: a new method of assigning patients to treatment and control groups. Clin Pharma and Therapeutics.

[49] Therneau TM (1993). How many stratification factors are “too many” to use in a randomization plan?. Control Clin Trials.

[50] Mustanski B, Moskowitz DA, Moran KO (2020). Evaluation of a Stepped-Care eHealth HIV Prevention Program for diverse adolescent men who have sex with men: protocol for a hybrid type 1 effectiveness implementation trial of SMART. JMIR Res Protoc.

[51] Mustanski B, Macapagal K, Li DH (2025). Effectiveness of the smart program: stepped-care HIV prevention for gay and bisexual adolescent boys. Health Psychol.

[52] (2023). Continuing to create age-appropriate ad experiences for teens meta soc technol co. Meta.

[53] Chandler JJ, Paolacci G (2017). Lie for a dime: when most prescreening responses are honest but most study participants are impostors. Soc Psychol Personal Sci.

[54] Song AV, Halpern-Felsher BL (2011). Predictive relationship between adolescent oral and vaginal sex: results from a prospective, longitudinal study. Arch Pediatr Adolesc Med.

[55] (2024). Data and statistics on adolescent sexual and reproductive health. HHS Office of Population Affairs.

[56] (2021). STI Screening Recommendations. CDC.

[57] Madkins K, Moskowitz DA, Moran K, Dellucci TV, Mustanski B (2019). Measuring acceptability and engagement of the keep it up! iIternet-based HIV prevention randomized controlled trial for young men who have sex with men. AIDS Educ Prev.

[58] Fisher JD, Fisher WA, Bryan AD, Misovich SJ (2002). Information-motivation-behavioral skills model-based HIV risk behavior change intervention for inner-city high school youth. Health Psychol.

[59] Golub SA, Kowalczyk W, Weinberger CL, Parsons JT (2010). Preexposure prophylaxis and predicted condom use among high-risk men who have sex with men. J Acquir Immune Defic Syndr.

[60] Parsons JT, Rendina HJ, Grov C, Ventuneac A, Mustanski B (2015). Accuracy of highly sexually active gay and bisexual men’s predictions of their daily likelihood of anal sex and its relevance for intermittent event-driven HIV pre-exposure prophylaxis. J Acquir Immune Defic Syndr.

[61] Mustanski B, Starks T, Newcomb ME (2014). Methods for the design and analysis of relationship and partner effects on sexual health. Arch Sex Behav.

[62] Crosby RA, Sanders SA, Yarber WL, Graham CA, Dodge B (2002). Condom use errors and problems among college men. Sex Transm Dis.

[63] Kalichman SC, Picciano JF, Roffman RA (2008). Motivation to reduce HIV risk behaviors in the context of the Information, Motivation and Behavioral Skills (IMB) model of HIV prevention. J Health Psychol.

[64] Kalichman SC, Cherry C, Browne-Sperling F (1999). Effectiveness of a video-based motivational skills-building HIV risk-reduction intervention for inner-city African American men. J Consult Clin Psychol.

[65] Carey MP, Schroder KEE (2002). Development and psychometric evaluation of the brief HIV Knowledge Questionnaire. AIDS Educ Prev.

[66] Szabo M, Lovibond PF (2022). Development and Psychometric Properties of the DASS-Youth (DASS-Y): an extension of the Depression Anxiety Stress Scales (DASS) to adolescents and children. Front Psychol.

[67] Badgett MVL, Baker KE, Conron KJ (2014). Best practices for asking questions to identify transgender and other gender minority respondents on population-based surveys (geniuss). https://williamsinstitute.law.ucla.edu/publications/geniuss-trans-pop-based-survey/.

[68] Mustanski B, Parsons JT, Sullivan PS, Madkins K, Rosenberg E, Swann G (2018). Biomedical and behavioral outcomes of keep it up!: an eHealth HIV prevention program RCT. Am J Prev Med.

[69] Newcomb ME, Sarno EL, Bettin E (2020). Relationship education and HIV prevention for young male couples administered online via videoconference: protocol for a national randomized controlled trial of 2GETHER. JMIR Res Protoc.

[70] Jafari M, Ansari-Pour N (2019). When and how to adjust your P values. Cell J.

[71] Gibbons RD, Hedeker D, Waternaux C, Davis JM (1988). Random regression models: a comprehensive approach to the analysis of longitudinal psychiatric data. Psychopharmacol Bull.

[72] Zeger SL, Liang KY, Albert PS (1988). Models for longitudinal data: a generalized estimating equation approach. Biometrics.

[73] Rubin DB (1974). Estimating causal effects of treatments in randomized and nonrandomized studies. J Educ Psychol.

[74] Rubin DB (1978). Bayesian inference for causal effects: the role of randomization. Ann Statist.

[75] Lee T, Shi D (2021). A comparison of full information maximum likelihood and multiple imputation in structural equation modeling with missing data. Psychol Methods.

[76] (2026). Dedoose: cloud application for managing, analyzing, and presenting qualitative and mixed method research data. Dedoose.

[77] Macapagal K, Matson M, Mustanski B (2019). Qual Mix Methods Data Anal Using Dedoose Pract Approach Res Soc Sci Thousand.

[78] Corbin J, Strauss A (2008). Techniques and Procedures for Developing Grounded Theory.

[79] Hsieh HF, Shannon SE (2005). Three approaches to qualitative content analysis. Qual Health Res.

[80] Miles MB, Huberman AM (1994). Qualitative Data Analysis: An Expanded Sourcebook.

[81] Morgan DL (1993). Qualitative content analysis: a guide to paths not taken. Qual Health Res.

[82] De Vries H, Elliott MN, Kanouse DE, Teleki SS (2008). Using pooled Kappa to summarize interrater agreement across many items. Field methods.

[83] Go VF, Morales GJ, Mai NT, Brownson RC, Ha TV, Miller WC (2015). Finding what works: identification of implementation strategies for the integration of methadone maintenance therapy and HIV services in Vietnam. Implementation Sci.

[84] Waltz TJ, Powell BJ, Chinman MJ (2014). Expert recommendations for implementing change (ERIC): protocol for a mixed methods study. Implement Sci.

[85] (2019). Security guide. Zoom Video Communications Inc.

[86] Proctor E, Silmere H, Raghavan R (2011). Outcomes for implementation research: conceptual distinctions, measurement challenges, and research agenda. Adm Policy Ment Health.

[87] Damschroder LJ, Lowery JC (2013). Evaluation of a large-scale weight management program using the consolidated framework for implementation research (CFIR). Implement Sci.

[88] Damschroder LJ, Aron DC, Keith RE, Kirsh SR, Alexander JA, Lowery JC (2009). Fostering implementation of health services research findings into practice: a consolidated framework for advancing implementation science. Implementation Sci.

[89] Smith JD, Li DH, Rafferty MR (2020). The implementation research logic model: a method for planning, executing, reporting, and synthesizing implementation projects. Implementation Sci.

[90] Ventuneac A, Li DH, Mongrella MC (2020). Exploring potential implementation barriers and facilitators of the SMART program: a stepped-care package of eHealth HIV prevention interventions for adolescent men who have sex with men. Sex Res Soc Policy.

[91] Morris ZS, Wooding S, Grant J (2011). The answer is 17 years, what is the question: understanding time lags in translational research. J R Soc Med.

[92] Liem WW, Casline E, Lorenzo J (2026). Enhancing LGBTQ+ inclusivity in an AI-powered sexual health chatbot: user-centered design approach through a nonprofit and academic partnership. J Med Internet Res.

[93] Perry RCW, Kayekjian KC, Braun RA, Cantu M, Sheoran B, Chung PJ (2012). Adolescents’ perspectives on the use of a text messaging service for preventive sexual health promotion. J Adolesc Health.

[94] Steinke J, Root-Bowman M, Estabrook S, Levine DS, Kantor LM (2017). Meeting the needs of sexual and gender minority youth: formative research on potential digital health interventions. J Adolesc Health.

[95] Fisher CB, Fried AL, Desmond M, Macapagal K, Mustanski B (2017). Facilitators and barriers to participation in PrEP HIV prevention trials involving transgender male and female adolescents and emerging adults. AIDS Educ Prev.

[96] Johnston CD, O’Brien R, Côté HCF (2023). Inclusion of women in HIV research and clinical trials. AIDS.

[97] Johnson MS, Adams VM, Byrne J (2024). Addressing fraudulent responses in online surveys: Insights from a web‐based participatory mapping study. People and Nature.

[98] Collins LM, Murphy SA, Strecher V (2007). The multiphase optimization strategy (MOST) and the sequential multiple assignment randomized trial (SMART): new methods for more potent eHealth interventions. Am J Prev Med.

