# Supplementary material for: Evaluating a Multimedia Messaging Intervention to Increase Adolescent HIV Testing: Protocol for a Hybrid Type 1 Effectiveness Implementation Study
Source: JMIR Res Protoc. 2026 May 18;15:e88465. doi: 10.2196/88465 (PMC13182879; doi:10.2196/88465)
Supplement: Peer Review Report 1 [file resprot-v15-e88465-s002.pdf]

1R01MH129207-01 Macapagal, Kathryn

EARLY STAGE INVESTIGATOR
NEW INVESTIGATOR

RESUME AND SUMMARY OF DISCUSSION: This application proposes to conduct a hybrid type 1 effectiveness-implementation study to test the effectiveness of Guy2Guy (G2G), a HIV prevention intervention for cisgender adolescent sexual minority males (ASMM), that has been adapted for transgender and gender diverse teens (TGD) and updated with new HIV prevention information about 4th generation HIV tests, Undetectable = Untransmittable, and pre-exposure prophylaxis. The adapted G2G intervention will be tested for effectiveness with 360 sexually active ASMM/TGD (ages 13-18) and the barriers/facilitators to the implementation of the intervention will be evaluated by a group of experts in ASMM/TGD research. The primary effectiveness outcome is objective evidence of an HIV test result (e.g., a photo of the result on the test device). This application builds upon strong pilot data and is supported by a strong team with the expertise needed to carry out the proposed study. The committee noted a few relatively minor weaknesses in the proposed study including the qualitative analytic plan, the attention matching procedures and the knowledge assessment. These weaknesses served to slightly limit the committee's otherwise high enthusiasm for the many strengths of this application.

DESCRIPTION (provided by applicant): Adolescent sexual minority males, transgender girls, and gender diverse teens (ASMM/TGD; ages 13-18) are disproportionately affected by HIV, accounting for 79% of new infections among this age group. Although CDC and USPTF recommend HIV screening among teens, testing rates among ASMM/TGD are very low (1 in 4 ASMM; <1 in 10 TGD). Increasing HIV testing in ASMM/TGD is critical in mitigating transmission rates and in linkage to care for those who test positive. Despite this alarming public health issue, few developmentally tailored HIV prevention interventions exist for ASMM/TGD, and to date, no CDC best-evidence interventions for this group are focused on increasing HIV testing as a primary outcome. G2G was a 6-week text message-based HIV risk behavior reduction program originally designed for ASMM. Active treatment arm content focused on providing HIV prevention information, increasing HIV prevention motivation, and increasing HIV prevention self- efficacy relative to an information-only healthy lifestyle control arm. The G2G pilot RCT showed adolescents in the active treatment arm were >3x more likely to report being tested for HIV at follow-up versus those in the control arm, even though only a minority of messages focused on testing. We propose to update G2G with the latest HIV prevention and testing science, make content inclusive of TGD, and test its effectiveness on the outcome of validated HIV testing (e.g., photo of test result). Moreover, widespread adoption of digital HIV prevention programs has lagged due to lack of attention to implementation throughout software development, intervention design, and testing. For instance, who should implement digital HIV prevention/testing interventions for teens (e.g., direct-to-consumer, clinics) remains an open question, and involving potential implementers in the development process can ensure intervention success; best practices for updating content and technology for digital HIV prevention/testing programs to prevent their obsolescence are lacking; and there is a lack of consensus on how best to reach SGM adolescents for such programs. We propose a Hybrid Type 1 trial that will establish G2G effectiveness and identify best practices for implementing digital HIV prevention/testing for teens via three specific aims. In Aim 1 we will collaborate with a youth advisory council to add content reflecting our increased emphasis on HIV testing and update gender inclusivity and cultural relevance of existing content. With prospective implementers' input, we will also modernize the G2G software platform with an eye toward implementation. In Aim 2 we will test G2G effectiveness on HIV testing in a nationwide randomized controlled trial with 360 ASMM/TGD aged 13-18. Finally, Aim 3 will convene a experts in ASMM/TGD youth research and service provision to identify and achieve consensus in processes of, requirements for, and barriers/facilitators to reaching and engaging ASMM/TGD for HIV testing/prevention. The goal of Aim 3 is to inform, develop, and disseminate

concrete tools to ensure HIV prevention programs reach ASMM/TGD. The panel will also recommend potential implementation models and adaptations needed to scale up G2G in real world settings.

PUBLIC HEALTH RELEVANCE: Despite recommendations for annual HIV screening among adolescents, testing rates among adolescent sexual minority males, transgender girls, and gender diverse teens (ASMM/TGD) are extremely low, and interventions specifically aimed at increasing adolescent testing rates are scarce. This proposal aims to test the effectiveness of a text message-based intervention on HIV testing behaviors and investigate factors that may facilitate widespread implementation of such programs among ASMM/TGD. Ultimately, increased HIV testing in this group will mitigate transmission rates and improve the HIV prevention and care continua in this population.

CRITIQUE 1

Significance: 3
Investigator(s): 1
Innovation: 3
Approach: 3
Environment: 1

Overall Impact: The proposed R01 addresses a significant public health issue – increasing HIV testing among adolescent sexual minority males and transgender and gender diverse teens. Overall, the scientific premise and rigor are strong based on the prior work supporting intervention's efficacy for HIV testing among a similar group and gaps in current intervention for the target population. The study is moderately innovative in the thoughtful approach to intervention adaptation and implementation. The team is well situated to do this work and complementary expertise in intervention and implementation science. Key strengths of the proposal include: strong research team and environment, preliminary work in this area with noted ability to recruit and retain ASMM and gender diverse youth, inclusion of a youth advisory board to guide adaptation, utilization of implementation science frameworks (e.g., CFIR), intervention that includes strategies to connecting youth to prevention and care services, and comprehensive data analysis plan. The few noted weaknesses are specific to the qualitative components of the study – the pre-implementation interviews and the analytical plan. It is also not clear if the intervention will be delivered in Spanish despite suggesting 30% Latino enrollment (for Aim 2 RCT). Overall, this is a strong application that has the potential to have a moderate to high impact on the field.

1. Significance:

Strengths

- Focus on adolescent SGM for improved HIV testing given disproportionate burden of disease and few interventions focused on this population. (major)
- Use of ADAPT-ITT and Trials for Intervention Principles for G2G intervention adaptation. (major)
- Use of effectiveness-implementation (Hybrid Type 1) design is useful for more rapid implementation, if warranted. (major)

Weaknesses

- While HIV testing is a crucial step in HIV prevention, connection to prevention and care services is also important and should be centered in this work. (moderate)

- Additional rationale for the combination of young men who have sex with men and transgender/gender diverse teens is warranted. (moderate)
- Some inconsistencies in description of prior work (e.g., G2G as 5 or 6 week program). (minor)

2. Investigator(s):

Strengths

- Very strong team, led by ESI, Dr. Macapagal. (major)
- Solid senior researchers to support ESI PI in leading large projects (e.g., Dr. Mustanski). (major)

Weaknesses

- None noted.

3. Innovation:

Strengths

- Thoughtful and innovative ç considerations. (major)

Weaknesses

- Other innovations notes (e.g., testing) are more significant than innovative.

4. Approach:

Strengths

- Strong basis of preliminary work, including G2G development and discussion with Youth Advisory Council. (major)
- Lots of experience recruiting and retaining young SGM in observational, intervention, and implementation research. (major)
- Use of hybrid type 1 effectiveness-implementation study design with qualitative pre-implementation components. (major)
- Open source platform increases implementation capacity, if warranted post testing. (moderate)
- Plans for prevention and care service linkage for HIV and other STIs are included. (major)
- Quantitative data analysis plan is well developed. (major)
- Aim 3's sequential mixed methods process focused on implementation challenges and facilitators will improve implementation and scale up of HIV related digital interventions. (major)

Weaknesses

- More information on how pilot G2G group used the intervention and confidentiality of text messages with this type of content to adolescents likely living at home (pilot group was 14-18 years old). (major)
- More detail about the pre-implementation interviewees (e.g., geographical region, identity: gender/sexual orientation) and who the youth beta testers (both rounds) are would be helpful to understand differences in intervention assessment and implementation needs. (major)
- Some additional information about a new HIV diagnosis and issues surrounding care linkage (especially for this age group) are warranted. (moderate)

- Qualitative evaluation data analysis plan is underdeveloped. (major)
- The rationale for not applying MOST is not compelling, especially given the adaptation process and expanded inclusion criteria. (moderate)

5. Environment:

Strengths

- Strong research environment at NU, with existing youth advisory boards. (major)

Weaknesses

- None noted.

Study Timeline:

Strengths

- Detailed and includes all major milestones.

Weaknesses

- None noted.

Protections for Human Subjects:

Acceptable Risks and/or Adequate Protections

- Human subjects monitoring is adequately addressed.

Data and Safety Monitoring Plan (Applicable for Clinical Trials Only):

Acceptable

- o inclusion of a DSMB for the study.

Inclusion Plans:

- Sex/Gender: Distribution justified scientifically
- Race/Ethnicity: Distribution justified scientifically
- For NIH-Defined Phase III trials, Plans for valid design and analysis: Not applicable
- Inclusion/Exclusion Based on Age: Distribution justified scientifically
- Inclusion/exclusion criteria scientifically justified.
- Analyses plan is well developed for the quantitative data; more is needed for the qualitative analyses.

Vertebrate Animals:

Not Applicable (No Vertebrate Animals)

Biohazards:

Not Applicable (No Biohazards)

Resource Sharing Plans:
Acceptable

Budget and Period of Support:
Recommend as Requested

CRITIQUE 2

Significance: 2
Investigator(s): 1
Innovation: 2
Approach: 3
Environment: 1

Overall Impact: This R01 is to conduct a hybrid type 1 effectiveness-implementation study to update and test effectiveness and implementation of Guy2Guy (G2G), an intervention developed by the team to reduce condomless sex in cisgender adolescent sexual minority males in the US, to include ne scientific developments (4th generation HIV tests, U=U, PrEP), to ensure content is inclusive of transgender and gender diverse teens, and to document and disseminate best practices for reaching ASMM/TGD for digital HIV prevention/testing. The study builds on prior pilot work which demonstrated success with respect to increasing HIV testing amongst ASMM exposed to 8-10 daily text messages addressing theoretical targets f behavior change – HIV prevention information, motivation and behavior skills. The proposal is responsive to PA-20-144 and NOT-MH-20-035, is led by a strong team and will be supported by a strong research environment. While there are a few minor weaknesses, this is a strong application with potential for moderate to high impact.

1. Significance:

Strengths

- Focus on increasing HIV testing (primary outcome) amongst cisgender adolescent sexual minority males and transgender and gender diverse teens in the US. This is a high priority group given their vulnerability to HIV.
- Will explore effectiveness of the intervention on STI testing, PrEP uptake, condomless sex, and interaction with providers re HIV care.
- The study will contribute to a growing evidence base re HIV prevention interventions for ASMM/TGD populations
- Will explore options for reaching ASMM/TGD for HIV prevention/testing
- The focus on developing G2G with an eye towards scale up is an important strength

Weaknesses

- None noted by reviewer.

2. Investigator(s):

Strengths

- The team is established and has the expertise and experience required for the proposed study including expertise in ehealth intervention development, implementation science, mixed methods as well as expertise and experience working with ASMM/TGD populations
- The team will be supported by an adolescent and community advisory group who will give input on multiple aspects of the intervention

Weaknesses

- None noted by reviewer.

3. Innovation:

Strengths

- Several innovative aspects including its focus on improving rates of HIV testing amongst ASMM/TGD populations, integration of ADAPT-ITT and TIPs frameworks for intervention adaptation, incorporation of a chatbot – an improvement over static Q&A functions, and on implementation of the intervention.

Weaknesses

- None noted by reviewer.

4. Approach:

Strengths

- The proposed study builds on the team's prior work, building on strengths and addressing limitations of prior work
- Content of G2G is guided by IMB model of behavior change
- Incorporates various features that are engaging to users – quizzes, badges, messages etc
- Integrated use of ADAPT-ITT and TIPs frameworks to guide adaptation is a strength
- Multi-prong approach to recruitment is a strength
- Use of objective outcomes measurements is a strength – e.g. photo of test result
- Stated thresholds / expectations for testing (at least 1 x annually)
- CFIR will be used to guide evaluation of implementation

Weaknesses

- Knowledge assessment via online survey and self-report in regards to provider communication are not optimal
- Qualitative evaluation data analysis plan is underdeveloped

5. Environment:

Strengths

- Participating institutions offer the needed resources to support the proposed work

Weaknesses

- None noted by reviewer.

Study Timeline:

Strengths

- Plans for recruitment / retention are sound

Weaknesses

- None noted by reviewer.

Protections for Human Subjects:

Acceptable Risks and/or Adequate Protections

Data and Safety Monitoring Plan (Applicable for Clinical Trials Only):

Acceptable

Inclusion Plans:

- Sex/Gender: Distribution justified scientifically
- Race/Ethnicity: Distribution justified scientifically
- For NIH-Defined Phase III trials, Plans for valid design and analysis: Not applicable
- Inclusion/Exclusion Based on Age: Distribution justified scientifically

]

Vertebrate Animals:

Not Applicable (No Vertebrate Animals)

Biohazards:

Not Applicable (No Biohazards)

Resource Sharing Plans:

Acceptable

Budget and Period of Support:

Recommend as Requested

CRITIQUE 3

Significance: 3
Investigator(s): 2
Innovation: 5
Approach: 2
Environment: 1

Overall Impact: This R01 application from an early stage/new investigator proposes to update and adapt a text message-based intervention (Guy2Guy; G2G) to increase HIV testing behaviors among adolescent sexual minority males, transgender girls, and gender diverse teens (ASMM/TGD). Efficacy will be evaluated in a hybrid type 1 RCT and barriers/facilitators for implementing the intervention at scale will be explored using several implementation science constructs including CFIR. The preliminary data presented from the investigators' prior experience with G2G, the complementary expertise of the study team, the environment and resources at Northwestern, and the formative work with advisory youth councils and community are all strengths that suggest this work could be impactful, and further evaluation of G2G in a large trial as suggested here has merit. Several weaknesses are noted, however. The underlying premise that the original messaging of G2G, which was oriented towards adolescent sexual minority males (ASMM), will not reach transgender and gender diverse teens (TGD) seems to rest primarily on interviews with HIV service organizations. Also, the degree to which attention-matching will be accomplished in the control group who receives healthy lifestyle information-only is not discussed and the rationale for and adequacy of using rapid qualitative analysis for pre-implementation interviews is not apparent. On balance, the above strengths outweigh these weaknesses and suggest this project would have moderately high impact.

1. Significance:

Strengths

- HIV testing rates are low among sexual and gender minority adolescents, a population that is at particularly high risk for HIV infection, and approaches tailored to reach this uniquely challenging-to-reach population are needed.
- If the aims of this project are achieved, strong evidence for the efficacy of a modernized G2G intervention will result, and guidance for optimizing this intervention and other digital interventions should be available
- The alignment of the intervention with the unique social and developmental needs of the target population will not only inform the modernization of G2G but should yield more generalizable information for the adaptation of other interventions.

Weaknesses

- The underlying premise that the original messaging of G2G, which was oriented towards adolescent sexual minority males (ASMM), will not reach transgender and gender diverse teens (TGD) as effectively is not well developed and seems to rest primarily on interviews with HIV service organizations.

2. Investigator(s):

Strengths

- The PI brings appropriate expertise in ASMM/TGD sexual health and HIV prevention research that has used online approaches and mixed methods.
- The PI was involved in the development and evaluation of the G2G intervention.
- The PI has experience, as a co-investigator, with hybrid effectiveness- implementation trials of digital interventions for SGM youth.
- Co-investigators bring complementary strengths in technology development, teen recruitment, the conduct of randomized controlled trials, quantitative methods, and implementation sciences, and there is a strong track record of collaboration.

Weaknesses

- None noted.

3. Innovation:

Strengths

- The focus on increasing HIV testing among SGM teens appears to be novel.
- Modernizing the G2G intervention with newer technologies such as chatbots could pave the way for others.

Weaknesses

- Studying how to reach teens per se is not innovative.

4. Approach:

Strengths

- The plan for involving YAC and community members in the G2G content review is well thought out and likely to yield meaningful contextualization/adaption.
- Although high rates of objective HIV testing results are anticipated, for those who do not provide these data, self-reports will be obtained, and appropriate sensitivity analyses are planned.
- Detailed plans are in place for linkage to care following self-reports of testing.
- That aim 3 stands alone and can be accomplished independently from the RCT ensures solid output from this aim re: barriers and facilitators for reaching ASMM/TGD with digital HIV prevention/testing interventions.
- Supplementing the use of CFIR with cost data will help to inform potential adopters of G2G's scalability.

Weaknesses

- The rationale for and adequacy of using rapid qualitative analysis for pre-implementation interviews is not apparent.
- The degree to which attention-matching will be accomplished in the control group who receives healthy lifestyle information-only is not specified. It appears that they will not have access to interactive features, making it difficult to disentangle the effect of the content itself vs. the interactivity of the experimental group. This may be a reasonable trade-off to make, but it is not discussed.

5. Environment:

Strengths

- The Institute for Sexual and Gender Minority Health and Wellbeing (ISGMH) at Northwestern has an in-house behavioral intervention software development team.
- The Center for Prevention Implementation Methodology at Northwestern and the Third Coast Center for AIDS Research
- Community input will be sought through a Community Collaboration Board and ISGMH’s Youth Advisory Council

Weaknesses

- None noted.

Study Timeline:

Strengths

- Realistic assumptions, informed from past work, are taken into account.

Weaknesses

- None noted

Protections for Human Subjects:

Acceptable Risks and/or Adequate Protections

- Waiver of parental permission per 45 CFR 46.408(c) with additional protections (e.g. assessment of decisional capacity and safety detailed).

Data and Safety Monitoring Plan (Applicable for Clinical Trials Only):

Acceptable

- At least 3 members with diverse backgrounds and appropriate expertise who are not members of the research team.

Inclusion Plans:

- Sex/Gender: Distribution justified scientifically
- Race/Ethnicity: Distribution justified scientifically
- For NIH-Defined Phase III trials, Plans for valid design and analysis: Not applicable
- Inclusion/Exclusion Based on Age: Distribution justified scientifically
- appropriate

Vertebrate Animals:

Not Applicable (No Vertebrate Animals)

Biohazards:

Not Applicable (No Biohazards)

Resource Sharing Plans:

Acceptable

Budget and Period of Support:

Recommend as Requested

THE FOLLOWING SECTIONS WERE PREPARED BY THE SCIENTIFIC REVIEW OFFICER TO SUMMARIZE THE OUTCOME OF DISCUSSIONS OF THE REVIEW COMMITTEE, OR REVIEWERS' WRITTEN CRITIQUES, ON THE FOLLOWING ISSUES:

PROTECTION OF HUMAN SUBJECTS: ACCEPTABLE

INCLUSION OF WOMEN PLAN: ACCEPTABLE

INCLUSION OF MINORITIES PLAN: ACCEPTABLE

INCLUSION ACROSS THE LIFESPAN: ACCEPTABLE

COMMITTEE BUDGET RECOMMENDATIONS: The budget was recommended as requested.

Footnotes for 1 R01 MH129207-01; PI Name: Macapagal, Kathryn Rose

NIH has modified its policy regarding the receipt of resubmissions (amended applications).See Guide Notice NOT-OD-18-197 at https://grants.nih.gov/grants/guide/notice-files/NOT-OD-18-197.html. The impact/priority score is calculated after discussion of an application by averaging the overall scores (1-9) given by all voting reviewers on the committee and multiplying by 10. The criterion scores are submitted prior to the meeting by the individual reviewers assigned to an application, and are not discussed specifically at the review meeting or calculated into the overall impact score. Some applications also receive a percentile ranking. For details on the review process, see http://grants.nih.gov/grants/peer_review_process.htm#scoring.

MEETING ROSTER

HIV/AIDS Intra- and Inter-personal Determinants and Behavioral Interventions Study Section
Risk, Prevention and Health Behavior Integrated Review Group
CENTER FOR SCIENTIFIC REVIEW
HIBI
07/12/2021 - 07/13/2021

Notice of NIH Policy to All Applicants: Meeting rosters are provided for information purposes only. Applicant investigators and institutional officials must not communicate directly with study section members about an application before or after the review. Failure to observe this policy will create a serious breach of integrity in the peer review process, and may lead to actions outlined in NOT-OD-14-073 at https://grants.nih.gov/grants/guide/notice-files/NOT-OD-14-073.html, NOT-OD-15-106 at https://grants.nih.gov/grants/guide/notice-files/NOT-OD-15-106.html, and NOT-OD-18-115 at https://grants.nih.gov/grants/guide/notice-files/NOT-OD-18-115.html, including removal of the application from immediate review.

CHAIRPERSON(S)

KIPKE, MICHELE D, PHD
PROFESSOR
DEPARTMENTS OF PEDIATRICS
AND PREVENTIVE MEDICINE
KECK SCHOOL OF MEDICINE
UNIVERSITY OF SOUTHERN CALIFORNIA
LOS ANGELES, CA 90028

MEMBERS

BUTLER, LISA MICHELLE, PHD
ASSOCIATE RESEARCH PROFESSOR
INSTITUTE FOR COLLABORATION ON HEALTH,
INTERVENTION, AND POLICY
UNIVERSITY OF CONNECTICUT
STORRS, CT 06269

COMULADA, WARREN SCOTT, DRPH
ASSOCIATE PROFESSOR
DEPARTMENT OF PSYCHIATRY
AND BIOBEHAVIORAL SCIENCES
SCHOOL OF PUBLIC HEALTH
UNIVERSITY OF CALIFORNIA, LOS ANGELES
LOS ANGELES, CA 90024

DODGE, BRIAN MARK, PHD
PROFESSOR
DEPARTMENT OF APPLIED HEALTH SCIENCE
INDIANA UNIVERSITY SCHOOL OF PUBLIC HEALTH
BLOOMINGTON, IN 47405

GRAHAM, SUSAN MARIE, MD, PHD
PROFESSOR
DIVISION OF ALLERGY AND INFECTIOUS DISEASES
DEPARTMENTS OF MEDICINE AND GLOBAL HEALTH
SCHOOL OF MEDICINE
UNIVERSITY OF WASHINGTON
SEATTLE, WA 98104

GROV, CHRISTIAN, PHD
PROFESSOR AND CHAIR
DEPARTMENT OF COMMUNITY HEALTH
AND SOCIAL SCIENCES
SCHOOL OF PUBLIC HEALTH AND HEALTH POLICY
CITY UNIVERSITY OF NEW YORK
NEW YORK, NY 10027

HANSEN, NATHAN B, PHD
DEPARTMENT HEAD AND PROFESSOR
DEPARTMENT OF HEALTH PROMOTION AND BEHAVIOR
COLLEGE OF PUBLIC HEALTH
UNIVERSITY OF GEORGIA
ATHENS, GA 30602

HORVATH, KEITH JOSEPH, PHD
ASSOCIATE PROFESSOR
DEPARTMENT OF CLINICAL PSYCHOLOGY
SAN DIEGO STATE UNIVERSITY
SAN DIEGO, CA 92120

IWELUNMOR, JULIET, PHD
ASSOCIATE PROFESSOR
DEPARTMENT OF BEHAVIORAL SCIENCE AND
HEALTH EDUCATION
COLLEGE FOR PUBLIC HEALTH AND SOCIAL JUSTICE
ST. LOUIS UNIVERSITY
ST. LOUIS, MO 63104

JOHNSON, DAWN M, PHD *
ASSOCIATE PROFESSOR
DEPARTMENT OF PSYCHOLOGY
UNIVERSITY OF AKRON
AKRON, OH 44325

LIPPMAN, SHERI ANN, PHD *
ASSOCIATE PROFESSOR
CENTER FOR AIDS PREVENTION STUDIES
UNIVERSITY OF CALIFORNIA, SAN FRANCISCO
SAN FRANCISCO, CA 94143

LOVEJOY, TRAVIS IAN, PHD
ASSOCIATE PROFESSOR
DEPARTMENT OF PSYCHIATRY
SCHOOL OF MEDICINE
OREGON HEALTH AND SCIENCE UNIVERSITY
PORTLAND, OR 97239

MACDONELL, KAREN KOLMODIN, PHD *
ASSOCIATE PROFESSOR
DEPARTMENT OF FAMILY MEDICINE
AND PUBLIC HEALTH SCIENCES
SCHOOL OF MEDICINE
WAYNE STATE UNIVERSITY
DETROIT, MI 48202

MUESSIG, KATHRYN E, PHD *
ASSISTANT PROFESSOR
DEPARTMENT OF HEALTH BEHAVIOR
GILLINGS SCHOOL OF GLOBAL PUBLIC HEALTH
UNIVERSITY OF NORTH CAROLINA AT CHAPEL HILL
CHAPEL HILL, NC 27599

OWCZARZAK, JILL, PHD *
ASSOCIATE PROFESSOR
DEPARTMENT OF HEALTH, BEHAVIOR AND SOCIETY
BLOOMBERG SCHOOL OF PUBLIC HEALTH
JOHNS HOPKINS UNIVERSITY
BALTIMORE, MD 21205

PATEL, VIRAJ V, MD, MPH *
ASSOCIATE PROFESSOR
DEPARTMENT OF MEDICINE
ALBERT EINSTEIN COLLEGE OF MEDICINE
BRONX, NY 10461

RAEL, CHRISTINE TAGLIAFERRI, PHD *
ASSISTANT PROFESSOR
COLLEGE OF NURSING
UNIVERSITY OF COLORADO ANSCHUTZ MEDICAL CAMPUS
AURORA, CO 80045

RAMSEY, SUSAN E, PHD
ASSOCIATE PROFESSOR
DIVISION OF GENERAL INTERNAL MEDICINE
RHODE ISLAND HOSPITAL
BROWN UNIVERSITY
PROVIDENCE, RI 02903

RODRIGUEZ-DIAZ, CARLOS EMANUEL, PHD *
ASSOCIATE PROFESSOR
DEPARTMENT OF PREVENTION AND COMMUNITY HEALTH
MILKEN INSTITUTE SCHOOL OF PUBLIC HEALTH
THE GEORGE WASHINGTON UNIVERSITY
WASHINGTON, DC 20052

SAFREN, STEVEN A, PHD
PROFESSOR
DEPARTMENT OF PSYCHOLOGY
COLLEGE OF ARTS AND SCIENCES
UNIVERSITY OF MIAMI
CORAL GABLES, FL 33124

SICONOLFI, DANIEL, MPH, PHD *
BEHAVIORAL SCIENTIST
RAND CORPORATION
PITTSBURGH, PA 15213

SSEWAMALA, FRED M, PHD
PROFESSOR
INSTITUTE FOR PUBLIC HEALTH
BROWN SCHOOL
WASHINGTON UNIVERSITY
ST. LOUIS, MO 63130

STOCKMAN, JAMILA KINSHASA, PHD
PROFESSOR
DIVISION OF GLOBAL PUBLIC HEALTH
DEPARTMENT OF MEDICINE
SCHOOL OF MEDICINE
UNIVERSITY OF CALIFORNIA, SAN DIEGO
LA JOLLA, CA 92093

SULLIVAN, PATRICK SEAN, PHD
PROFESSOR
DEPARTMENT OF EPIDEMIOLOGY
ROLLINS SCHOOL OF PUBLIC HEALTH
EMORY UNIVERSITY
ATLANTA, GA 30322

TANNER, AMANDA E, MPH, PHD *
ASSOCIATE PROFESSOR
DEPARTMENT OF PUBLIC HEALTH EDUCATION
SCHOOL OF HEALTH AND HUMAN SCIENCES
UNIVERSITY OF NORTH CAROLINA GREENSBORO
GREENSBORO, NC 27402

THAMES, APRIL D, PHD
ASSOCIATE PROFESSOR
DEPARTMENT OF PSYCHOLOGY
UNIVERSITY OF SOUTHERN CALIFORNIA
LOS ANGELES, CA 90089

THIELMAN, NATHAN M, MD, MPH *
PROFESSOR
DEPARTMENT OF MEDICINE
DUKE UNIVERSITY SCHOOL OF MEDICINE
DURHAM, NC 27710

WEBEL, ALLISON R, PHD
PROFESSOR
SCHOOL OF NURSING
UNIVERSITY OF WASHINGTON
SEATTLE, WA 98195

WILSON, TRACEY ELIZABETH, PHD *
PROFESSOR
DEPARTMENT OF COMMUNITY HEALTH SCIENCES
SCHOOL OF PUBLIC HEALTH
DOWNSTATE MEDICAL CENTER
THE STATE UNIVERSITY OF NEW YORK
BROOKLYN, NY 11203

WILTON, LEO, PHD, MPH
PROFESSOR
DEPARTMENT OF HUMAN DEVELOPMENT
COLLEGE OF COMMUNITY AND PUBLIC AFFAIRS
BINGHAMTON UNIVERSITY
BINGHAMTON, NY 13902

WINDSOR, LILIANE CAMBRAIA, PHD
ASSOCIATE PROFESSOR
SCHOOL OF SOCIAL WORK
THE UNIVERSITY OF ILLINOIS AT URBANA-CHAMPAIGN
URBANA, IL 61801

YBARRA, MICHELE L., PHD, MPH *
CEO AND RESEARCH DIRECTOR
CENTER FOR INNOVATIVE PUBLIC HEALTH RESEARCH
SAN CLEMENTE, CA 92672

SCIENTIFIC REVIEW OFFICER

RUBERT, MARK P, PHD
SCIENTIFIC REVIEW OFFICER
CENTER FOR SCIENTIFIC REVIEW
NATIONAL INSTITUTES OF HEALTH
BETHESDA, MD 20892

EXTRAMURAL SUPPORT ASSISTANT

CAMBRELEN, AMY ANGELA
EXTRAMURAL SUPPORT ASSISTANT
CENTER FOR SCIENTIFIC REVIEW
NATIONAL INSTITUTE OF HEALTH
BETHESDA, MD 20892

* Temporary Member. For grant applications, temporary members may participate in the entire meeting or may review only selected applications as needed.

Consultants are required to absent themselves from the room during the review of any application if their presence would constitute or appear to constitute a conflict of interest.
